# Long noncoding RNA H19 promotes the acquisition of a mesenchymal-like invasive phenotype in mesothelial primary cells through an HDAC1-mediated WT1/Sp1 switch

**DOI:** 10.1038/s41419-025-07956-8

**Published:** 2025-08-31

**Authors:** Giulio Bontempi, Federica Michetti, Michela Terri, Cecilia Battistelli, Alice Conigliaro, Sabrina Garbo, Claudia Montaldo, Sergio Valente, Clemens Zwergel, Antonello Mai, Azadeh Nahavandi Araghi, Alessandro Domenici, Paolo Menè, Marco Tripodi, Raffaele Strippoli

**Affiliations:** 1 Department of Molecular Medicine, Sapienza University of Rome, Rome, Italy; 2 Gene Expression Laboratory, National Institute for Infectious Diseases, Lazzaro Spallanzani IRCCS, Rome, Italy; 3 Department of Biomedicine, Neurosciences and Advanced Diagnostics (Bi.N.D.), Section of Biology and Genetics, University of Palermo, Palermo, Italy; 4 Department of Drug Chemistry and Technologies, Sapienza University of Rome, Rome, Italy; 5 Escuela de Doctorado,, Universidad Autónoma de Madrid, Madrid, Spain; 6 Centro de Biología Molecular Severo Ochoa, CSIC-UAM, Madrid, Spain; 7 Renal Unit, Department of Clinical and Molecular Medicine, Sant’Andrea University Hospital, Sapienza University of Rome, Rome, Italy

**Keywords:** Non-coding RNAs, Epithelial-mesenchymal transition, Chronic inflammation

## Abstract

Peritoneal fibrosis is a pathological alteration of the peritoneal membrane occurring in pro-inflammatory conditions, including peritoneal dialysis (PD), a renal replacement therapy. Characteristic of this process is the acquisition of invasive/pro-fibrotic abilities by mesothelial cells (MCs) through induction of mesothelial to mesenchymal transition (MMT), a cell-specific form of EMT. Long noncoding (lnc) RNAs act as major players in physiologic regulatory circuitries of the cell. While LncRNA-H19 (lncH19), one of the first lncRNAs identified, has been broadly studied in tumorigenesis, its role in peritoneum fibrotic diseases has been scarcely addressed so far. Aim of this study was to investigate the role of H19 in the acquisition of a mesenchymal-like phenotype in primary fibrotic MCs from PD patients, and to elucidate epigenetic mechanisms controlling its expression. Genetic silencing/ectopic expression experiments revealed that H19 promoted the expression of MMT markers while downregulating the epithelial marker E-Cadherin, and favored MC directed migration and invasion on a collagen matrix. Silencing of three main H19 isoforms revealed a synergistic activity in the induction of a mesenchymal phenotype. Treatment with MS-275, an HDAC1-3 specific inhibitor previously known to promote MMT reversal, as well as HDAC1 genetic silencing, downregulated lncRNA H19 expression. Bioinformatic analysis revealed a binding sequence of Wilm’s Tumor Protein 1 (WT1), the master gene of mesothelial differentiation, on the H19 promoter at an area with multiple acetylation peaks partially overlapping the binding site of Specificity protein 1 (Sp1), another transcription factor active in cellular plasticity regulation. Genetic silencing and Chromatin Immunoprecipitation (ChIP) experiments demonstrated that HDAC1 inhibition promotes a switch between WT1 and Sp1 in H19 promoter occupancy, favoring an inhibitory effect of WT1 on H19 expression and the reversal towards an epithelial-like phenotype. Overall, we discovered an HDAC1-WT1/Sp1-H19 axis potentially relevant to the design of new therapies aimed at counteracting peritoneal fibrosis.

## Introduction

Mesothelial to mesenchymal transition (MMT) is a cellular process implicated in the embryogenesis of trunk organs and their cavities, as well as in the pathogenesis of chronic fibrotic pathologies of the serosal membranes of the body, such as peritoneal alterations during peritoneal dialysis (PD), chronic lung fibrosis, fibrotic adherences after abdominal surgery [1, 2]. Upon exposure to biomechanical, proinflammatory, and profibrotic stimuli, mesothelial cells (MCs) undergo a complex reprogramming process characterized by the acquisition of a mesenchymal-like proinflammatory phenotype and playing a role in the generation of fibrosis, angiogenesis, and vasculopathy of the peritoneal membrane in a complex network of extracellular signals generated by various cellular types, including leukocytes, stably residing or recirculating along the peritoneal membrane [3, 4]. Besides the functional alteration of the serosal membranes, peritoneal fibrosis offers the soil for the progression of tumors invading the peritoneal space, such as ovarian and colon carcinoma [5, 6]. Thus, the study of molecular mechanisms controlling MC plasticity has both a basic and translational relevance [7].

Histone acetyltransferases (HATs) and histone deacetylases (HDACs) enzymes control gene expression in eukaryotic cells due to alteration of chromatin structure. To date, 18 different human HDACs have been identified, and they have been categorized into four classes (I–IV) based on their similarity to Saccharomyces cerevisiae’s HDACs (RPD3, HDA1, and SIR2) [8, 9]. Class I HDAC expression is increased in both tumor and non-tumor inflammatory/profibrotic conditions, including peritoneal fibrosis [5, 10]. Moreover, HDAC class I activity was previously demonstrated as a major epigenetic mechanism orienting cells toward a mesenchymal-like profibrotic phenotype [11, 12]. We previously demonstrated that HDAC1-3 inhibition by MS-275 favors MMT reversal toward an epithelial-like phenotype [13, 14].

Several noncoding RNAs, including long noncoding (lnc) RNAs, have been shown to regulate the expression of genes important for cell plasticity and fibrosis both at a post-transcriptional level and by modifying the chromatin epigenetic landscape. LncRNAs act as major players in physiologic regulatory circuitries of the cell and are largely deregulated in several human diseases, such as tumors [15]. By interacting with DNA, RNA, and proteins, lncRNAs can influence the structure and function of chromatin, regulate the transcription of both nearby and distant genes, and impact RNA splicing, stability, and translation [16]. Additionally, lncRNAs play a role in the formation and regulation of organelles and nuclear condensates [17].

Specifically, one prominent lncRNA, LncRNA-H19 (H19), one of the first lncRNAs identified, has caught particular attention due to its role in tumorigenesis and cancer progression [18, 19]. H19 is predominantly active during fetal development, with the highest expression in developing skeletal and smooth muscles. H19 has a strong level of conservation across eutherian species and is expressed in humans in several isoforms [20].

Through its regulatory functions, H19 can modulate gene expression and signaling pathways, thus influencing key cellular processes, including epithelial to mesenchymal transition (EMT). The interplay between H19 and EMT highlights the intricate molecular mechanisms orchestrated by lncRNAs in driving pathological processes. The effect of H19 in EMT induction has been demonstrated to be cell-specific. Indeed, in hepatocellular carcinoma, H19 has been demonstrated to inhibit EMT due to induction of the antifibrotic miRNA miR200, leading to increased E-cadherin expression [21]. Conversely, in colon carcinoma, H19 promotes EMT via binding to hnRNPA2B1 [22]. In non-transformed endothelial cells, H19 favors endothelial to mesenchymal transition (EndMT) induction regulating the DNA demethylase TET1 at the post-transcriptional level [23].

H19 has recently been shown to be expressed in human and mouse mesothelium and to mediate peritoneal fibrosis [24, 25]. However, no information has been provided on specific isoforms, and the molecular mechanisms controlling both H19 expression and activity have been incompletely elucidated.

Aim of this study was to characterize the impact of H19 in the acquisition of a mesenchymal-like phenotype, the characterization of the role of specific isoforms expressed in human primary MCs from PD patients, as well as the epigenetic regulation of its expression.

## Materials and methods

### Patients, tissue samples, and cells

Effluent-derived MCs were isolated from clinically stable PD patients (Table 1). The bags with peritoneal dialysates from patients were left untouched for about 3 h to allow the deposition of floating cells at the bottom of the bags. With a sterile pipette, the supernatant was removed, leaving ~200 ml of sediment at the bottom of the bags. The cells were transferred in 50 ml tubes and centrifuged at 2000 rpm for 5 min. The cell pellets were suspended in 4 ml of culture medium and were seeded in 60 cm tissue culture dishes. The culture medium was replaced every 2 days, and the cells were washed to remove all detached peritoneal leukocytes. Effluent-derived MCs were cultured in Earle’s M199 supplemented with 10% FBS (GIBCO® Life Technology, Monza, Italy), 2 mM L-glutamine (EuroClone), 100 U/ml penicillin, 100 µg/ml streptomycin (Gibco-Life Technologies) and amphotericin B (2.5 µg/ml).

**Table 1:** List of PD patients enrolled in this study.

Patients	sex	age	cause of kidney failure	Diabetes	Hypertension	months on PD	PD technique	exchanges	PD solution	glucose (mg/dl)	d/p creat 4 h PET	peritonitis	hemoperitoneum	escapes	epitheliod/non-epitheliod
1	M	56	malignant hypertension	no	yes	40	CAPD	1	FMC stay-safe balance®	2270	0.48	no	no	no	E
2	M	69	unknown	no	yes	44	CAPD	4	FMC stay-safe balance®	1580	0.45	no	no	no	E
3	F	85	p-ANCA vasculitis	no	yes	58	CAPD	3	FMC stay-safe balance®	1815	0.5	no	no	no	NE
4	M	75	Ig A GNF	no	yes	92	APD	15 liters/night	FMC stay-safe balance®	1360	0.67	no	no	no	NE
5	M	61	diabetes/hypertension	yes	yes	12	CAPD	1	FMC stay-safe balance®	2270	0.63	no	no	yes	E
6	F	66	p-ANCA vasculitis	no	yes	31	CAPD/CCPD	4/20 liters/24 h	FMC stay-safe balance®	2270	0.66	no	no	no	E
7	M	66	Chronic pielonephritis	no	yes	72	CAPD	2	FMC stay-safe balance®	2270	0.66	2	no	no	NE
8	M	53	ADPKD	no	yes	6	CAPD	3	FMC stay-safe balance®	2270	0.5	2	no	yes	E
9	M	54	unknown	no	yes	13	CAPD	1	FMC stay-safe balance®	1360	0.71	no	no	no	E
10	M	64	Ig A GNF	no	yes	55	CCPD	25 liters/24 h	FMC stay-safe balance®	1580	0.68	2	no	yes	NE
11	M	57	Type 1 diabetes mellitus	yes	yes	56	CCPD	20 liters/24 h	FMC stay-safe balance®	1815	0.54	no	no	no	E

The human mesothelial cell line MeT-5A (ATCC, Rockville, MD) was cultured in Earle’s M199 as above but without amphotericin B; these cells were isolated from pleural fluids obtained from non-cancerous individuals. Effluent-derived MCs, human peritoneal mesothelial cells (HPMCs), and MeT-5A cell lines were grown at 37 °C in a humidified atmosphere with 5% CO2. In some experiments to enhance MMT-like features, effluent-derived MCs and Met-5A were treated with TGFβ1 (2 ng/ml). The cytokine dose used is in the range of those detected in peritoneal-dialysis fluids from patients with peritonitis and is similar to those used in previous studies) [14].

Experiments on effluent-derived MCs were performed according to guidelines from the ethics committee of Sant’Andrea Hospital, Sapienza University (Rome, Italy). Written informed consent was obtained from all PD patients. The protocol and informed consent were reviewed and approved by the Ethics Committee of Clinic Investigation of Sapienza University ref: 4697_2017 (Rome, Italy).

### Antibodies and chemicals

Anti-SNAIL (L70G2) mAb was from Cell Signaling Technology (Danvers, MA); anti-PAI-1 (sc-5297), -TUBULIN (sc-32293), -HSP90 (sc-13119), -GAPDH (sc-32233) mAbs were from Santa Cruz Biotechnology (Dallas, TX). Rabbit polyclonal antibody (pAb) anti-TGF-β Receptor Antibody type I (ABF17-I) was from Merck Life Science S.r.l (Darmstadt, Germany); pAb anti-WT1 (12609-I-AP) was from Proteintech (Chicago, IL); pAbs anti-SMAD2/SMAD3 (31025) were from Cell Signaling Technology. Wheat germ agglutinin (w11261) was from Thermo Fisher Scientific (Waltham, MA). MS-275, used at the concentration of 250 nM, was from the Mai laboratory.

### Western blotting

Monolayers of effluent-derived MCs or MeT-5A cells were lysed in Laemli SDS sample buffer, reducing (Tris 60 µM pH 6.8, 2% SDS, 10% glycerol, 5% 2-β mercaptoethanol), were boiled for 5’ at 95 °C, and were loaded on 12% acrylamide gels. For subcellular fractionation, the cells were lysed with an appropriate buffer (10 mM HEPES, pH 7.6, 10 mM KCl, 0.1 mM EDTA, 0.1 mM EGTA, 0.5 mM DTT, 100 mM phenylmethylsulfonyl fluoride, protease inhibitor cocktail [Roche], and 0.05% NP-40). Nuclear-cytoplasmic fractionations were separated by centrifugation. Cytosolic fraction was precipitated with acetone, and nuclei were lysed with another buffer (20 mM Hepes, pH 7.6, 0.4 M NaCl, 1 mM EDTA, 1 mM EGTA, 1 mM DTT, 0.75 mM spermidine, 0.15 mM spermine,100 mM phenylmethylsulfonyl fluoride, and protease inhibitor cocktail [Roche]) and centrifuged at 13000 rpm to remove the DNA. Both fractions were eluted with sample buffer and analyzed by western blotting. Gels were electrophoresed at 100 V in Running Buffer (25 mM Tris, 190 mM glycine; 0.1% SDS) and then transferred to a nitrocellulose membrane (Pure Nitrocellulose Membrane 0.45 μm; Bio-Rad, Hercules, CA) at 100 V for 1 h and 30’ in Transfer Buffer (50 mM Tris, 40 mM glycine; 0.1% SDS; 20% Methanol). Blots were blocked in 5% non-fat milk prepared in TBS-Tween (10 mM Tris-HCl pH 7.5; 150 mM NaCl; 0.05% Tween 20) and incubated overnight with the primary antibody. The day after, the blots were incubated with HRP- conjugated species-specific secondary antibodies (Goat Anti-Mouse IgG (H + L) HRP Conjugated 170-6516 or Goat Anti-Rabbit IgG (H + L)-HRP Conjugated 172-1019, Bio-Rad). Nitrocellulose-bound antibodies were detected by chemiluminescence with ECL (WESTAR Nova 2.0, Cyanagen, WESTAR etaC, Cyanagen), and the signal was revealed through autoradiography X-ray film. Raw data of WB shown in this study are available in the supplementary material (Fig. S1).

### Reverse-transcriptase polymerase chain reaction

mRNAs extracted from cell cultures with miRNeasy Mini Kit (QIAGEN) were reverse transcribed with SuperScript™ IV VILO™ Master Mix. cDNAs were amplified by qPCR reaction using GoTaq® qPCR Master Mix (Promega, Madison, WI, USA), and the reaction was carried out in BioRad-iQ-iCycler. The results were analyzed with CFX Manager software (Biorad), and the relative amounts obtained with the 2^(−ΔCt)^ method were normalized with respect to the gene L34.

The specific primer pairs are listed in Table 2.

**Table 2:** qRT-PCR primers used in this study.

Name	Sequences
L34	F 5′-GTCCCGAACCCCTGGTAATAG-3′ R 5′-GGCCCTGCTGACATGTTTCTT-3′
TGFβRI	F 5′-AACTTCCAACTACTGGCCCT-3′ R 5′-GGTGAATGACAGTGCGGTTG-3′
TGFβ1	F 5′-AAGGACCTCGGCTGGAAGGTG-3′ R 5′-CCCGGGTTATGCTGGTTGTA-3′
SMAD2	F 5′-ACAGCTAGGCAGGGCAACTA-3′ R 5′-GGGCAGAGTTCACAGTCACA-3′
SMAD3	F 5′-CCCCAGAGCAATATTCCAGA-3′ R 5′-GACATCGGATTCGGGGATAG-3′
PAI-1	F 5′-AGTGGACTTTTCAGAGGTGGA-3′ R 5′-GCCGTTGAAGTAGAGGGCATT-3′
E-CADHERIN	F 5′-TACGCCTGGGACTCCACCTA-3′ R 5′-CCAGAAACGGAGGCCTGAT-3′
OCCLUDIN	F 5′-AAGGTCAAAGAGAACAGAGCAAGA-3′ R 5′-TATTCCCTGATCCAGTCCTCCTC-3′
HDAC1	F 5′-CATCGCTGTGAATTGGGCTG-3′ R 5′-CCCTCTGGTGATACTTTAGCAGT-3′
COL1A1	F 5′-AGCCAGCAGATCGAGAACAT-3′ R 5′-TCTTGTCCTTGGGGTTCTTG-3′
FN1	F 5′-GGCTGACAGAGAAGATTCCCG-3′ R 5′-AGCTGGGTCTGCTAACATCAC-3′
MMP14	F 5′-TCTGGCGGGTGAGGAATA-3′ R 5′-CTCTCGTAGGCAGTGTTGA-3′
α-SMA (ACTA2)	F 5′-CAGCCAAGCACTGTCAGG-3′ R 5′-CCAGAGCCATTGTCACAC-3′
H19 (Variant 1-2-3)	F 5′-GCCTTTGAATCCGGACACAA-3′ R 5′-GCTGTTCCGATGGTGTCTTT-3′
H19 (Variant 1-2)	F 5′-GAGGATGGTGCAGGCAGG-3′ R 5′-TCCTGCTTGTCACGTCCAC-3′
H19 (Variant 3)	F 5′-AGTTGAAGCCAGGTCTCCAG-3′ R 5′-CTCTGTCCTCGCCGTCAC-3′
H19 Promoter segment n1	F 5′- AAGGTCCAGAGGGAGACCCT -3′ R 5′- ACGTTTCTGTGGGTGAACCC -3′
H19 Promoter segment n2	F 5′- GGGTTCACCCACAGAAACGT -3′ R 5′- CAACCGATTCTGCACCATC -3′
H19 Promoter segment n3	F 5′- GATGGTGCAGAATCGGTTG -3′ R 5′- TCAGACACGTAGCCCAATGT -3′
H19 Promoter segment n4	F 5′- TTCCCCTTCTGTCTCACCAC -3′ R 5′- TCCCATGAGCGTCCTATTCC -3′

### Chromatin immunoprecipitation assay (ChIP)

ChIP analysis was performed as previously reported [14]. 5 μg of anti-WT1/SP1/H3K27Ac or rabbit IgG were used. After washing, samples were eluted with the elution buffer (NaHCO3 100 mM, SDS 1%) and treated with 10 μg of RNase A and 240 μg of proteinase K (Sigma-Aldrich). The extracted DNA was used in the qPCR analyses. Specific primer pairs used are shown in Table 2. Data were expressed as (IP-IgG)/Input. The analysis of WT1 binding sites on the H19 promoter was performed by PROMO software using version 8.3 of TRANSFAC. The analysis of acetylation peaks on H19 was provided by ENCODE using the UCSC Genome Browser on Human (GRCh37/hg19).

### siRNA-mediated knockdown and ectopic expression

100 × 10^3^ MCs were seeded on 12-well plates 24 h prior to transfection. Cells were transfected with either 100 pmol of Silencer® Select (CAT. 4390816) against human H19, SP1, and WT1 or the same amount of ON-TARGETplus Non-targeting siRNA #1 (Cat. D-001810-01-50) and 2 μl Lipofectamine® RNAiMAX Reagent from Thermo Fisher Scientific (Waltham, MA, USA) in 200 μl Optimem from Gibco (Waltham, MA, USA). 1 ml of supplemented medium per well was also added. The transfection lasted 48 h. Knockdown efficiency was determined by RT-PCR and/or western blot.

H19 siRNAs used in this study were: silencer select siRNA (all variants) H19 Catalog# 4390816 Assay ID n272446 from Thermo Fisher;

custom Silencer select siRNA (Variant 1-2) S GAGUUAGCAAAGGUGACAUCUUCTCdTdT AS GAGAAGAUGUCACCUUUGCUAACUCUCdTdT;

custom Silencer select siRNA (Variant 3) S GUUCUGAGGUGAUCAUGACUGGGACdTdT AS GUCCCAGUCAUGAUCACCUCAGAACACdTdT.

To ectopically express H19, 10 × ${10}^{4}$ MCs were seeded in 12-well plates 24 h prior to transfection. H19-overexpressing cells were obtained by transient transfection with pFLAG-CMV-1 H19 (gift from Prof. Alice Conigliaro) or the empty vector by Lipofectamine 2000 transfection reagent (Invitrogen, Thermo Fisher) according to the manufacturer’s protocol. Cells were collected 48 h after transfection.

### Confocal microscopy and immunofluorescence

For H19 staining, ViewRNA™ Cell Plus Assay Kit by ThermoFisher (Catalog number: 88-19000-99) was used according to the manufacturer’s instructions. In particular, H19 ViewRNA Cell Plus Probe Set 647 Cat. VX-01, Assay ID VA6-3167844-VC, was used. Coverslips were mounted in Prolong Gold antifade (Life Technologies) and examined under a confocal microscope (Carl Zeiss, Cell Discoverer 7, Germany). Digital images were acquired with the ZenBlue software. A minimum of 4 fields per sample from three independent experiments was analyzed.

### Migration and Invasion assay

For migration assay, primary MCs from patients undergoing PD were allowed to reach 100% confluency in an 12 multiwell plate. MCs were genetically silenced for H19 in the presence of TGFβ1 or for 48 h in culture medium supplemented with 10% FCS. Therefore, a scratch was performed with a 10 μl tip, and after 18 h, cells were fixed and stained.

For transwell invasion assays, 8-μm pore. 6.5 mm Insert 24-well cell-culture plates (Corning Inc.) coated with type I collagen (0.1 mg/mL; Upstate Biotechnology) were used. 12.5 × 10^3^ MeT5A cells were plated in the upper chamber in serum-reduced medium (1% FBS); in the lower chamber, the M199 medium was supplemented with 20% FBS as a chemoattractant. Cells were fixed with 100% MeOH and stained with Crystal violet solution. MCs were allowed to invade for 5 h.

### Statistical analysis

Statistical significance was determined with a *t*-test (one-tailed) using GraphPad Prism version 8.0 (La Jolla, CA, USA). Differences were considered significant *: *P* < 0.05; ** *P* < 0.01; *** *P* < 0.001.

## Results

### H19 is expressed in three variants in primary MCs from PD patients

**Fig. 1:**
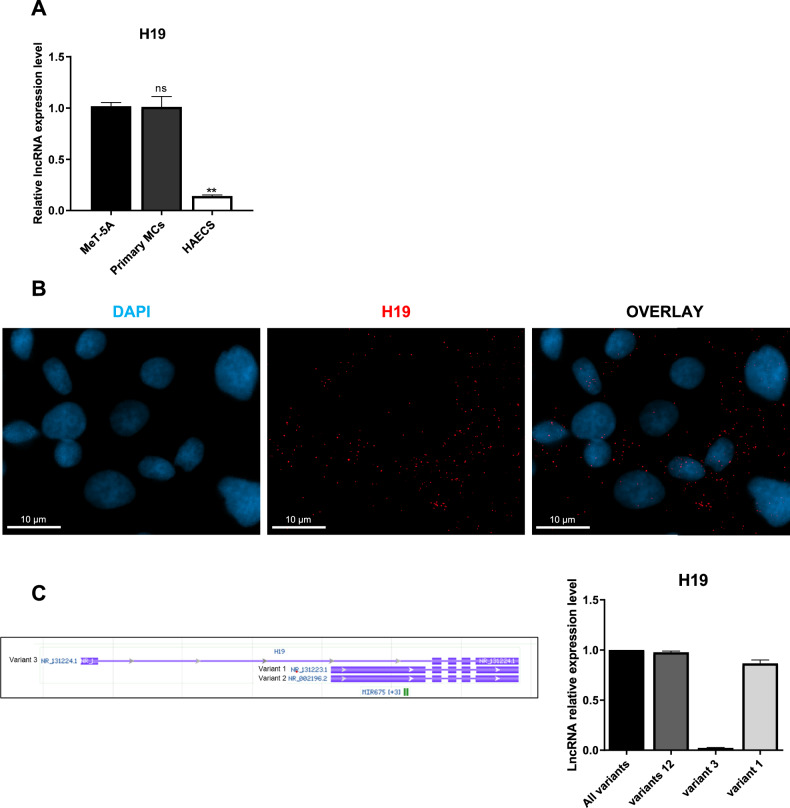
H19 is expressed in three variants in MCs from PD patients. **A** RT-qPCR experiment showing H19 expression from total RNA of mesenchymal-like MeT5A cells, MCs from PD patients and HAECs. L34 mRNA levels were used for normalization. Bars represent means ± SEM of 3 experiments. **B** Representative image of H19 expression (red), in primary MCs by FISH. Nuclei (blue) are stained with DAPI. Representative images are shown from one experiment of 3 performed. **C** Image showing the sequence of the three main H19 isoforms from Genome Browser and RT-qPCR experiment showing the expression of the H19 isoforms 1-3. L34 mRNA levels were used for normalization. Bars represent means ± SEM of 4 experiments.

The expression of H19 in human MCs was first investigated. H19 was found expressed in the MeT5A cell line and in primary MCs from PD patients at a higher level compared to Human Aortic Endothelial Cells (HAECS), where it was previously reported [23, 26] (Fig. 1A).

Furthermore, in order to visualize H19 expression and obtain insight on its cellular localization, RNA FISH experiments were performed and revealed a distribution in small dots in untreated primary MCs (Fig. 1B).

The expression of H19 main variants was then studied. Genome Browser analysis of H19 revealed the presence of three different variants: two are very similar to each other, while the third one is shorter due to the presence of an alternative exon (Fig. 1C, left). qRT-PCR analysis using a specific probe for each variant revealed that while variants 1 is predominant, variants 2 and 3 (as speculated, with respect to variant 2, by the comparison between oligos recognizing 1 and recognizing 1-2 variants) are by far less expressed (Fig. 1C, right).

### H19 variants favor the acquisition of a mesenchymal-like phenotype in MCs

**Fig. 2:**
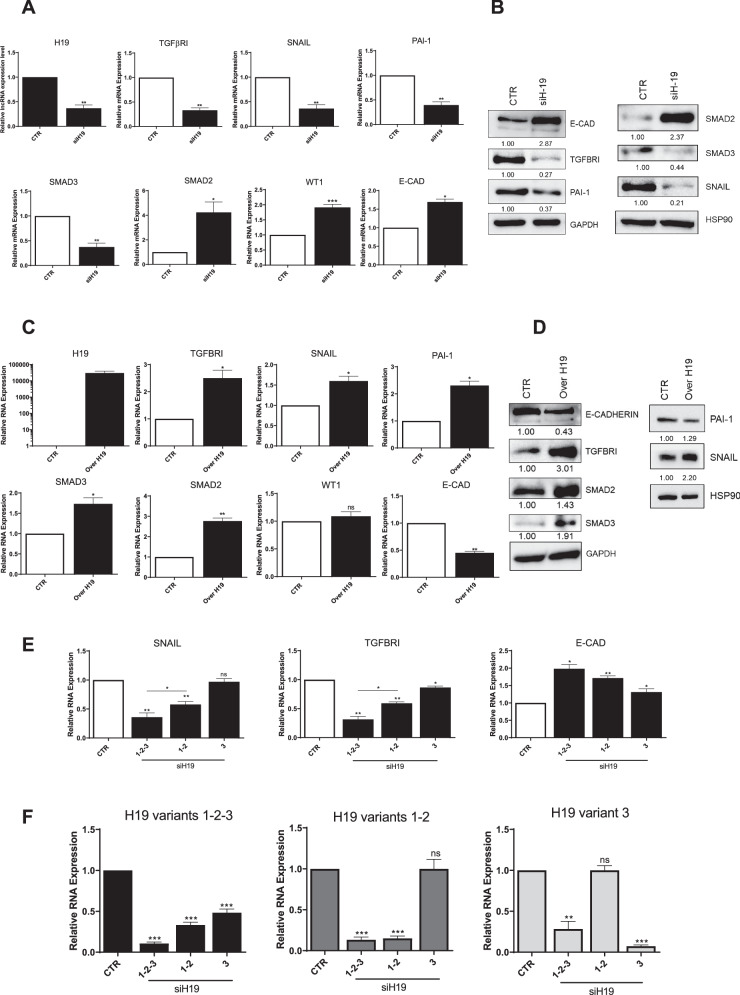
H19 variants favor the acquisition of a mesenchymal-like phenotype in MCs. **A** RT-qPCR showing the expression of H19, TGFBRI, SNAIL, PAI-1 (top) SMAD2, SMAD3, WT1, and E-CAD (bottom) from H19 genetically silenced MCs from PD patients. L34 mRNA levels were used for normalization. Bars represent means ± SEM of 4 experiments. **B** Western Blot (WB) experiment showing the expression of E-CAD, TGFBRI, PAI-1, GAPDH (left). SMAD2, SMAD3, SNAIL (right) in H19 genetically silenced MC from PD patients. GAPDH and HSP90 were used for normalization, respectively. Representative images are shown from one experiment of 3 performed. **C** RT-qPCR showing the expression of H19, TGFBRI, SNAIL, PAI1 (top) SMAD2, SMAD3, WT1, E-CAD (bottom) from H19 ectopically expressing MCs from PD patients. L34 mRNA levels were used for normalization. Bars represent means ± SEM of 4 experiments. **D** Western Blot (WB) experiment showing the expression of E-CAD, TGFBRI, SMAD2, SMAD3, PAI-1, and Snail in H19 ectopically expressing MC from PD patients. GAPDH and SHP90 were used for normalization. Representative images are shown from one experiment of 3 performed. **E** RT-qPCR showing the expression of SNAIL, TGFBRI, and E-CAD from 1–3, 1–2, and 3 specific H19 variant genetically silenced MCs from PD patients. L34 mRNA levels were used for normalization. Bars represent means ± SEM of 4 experiments. **F** RT-qPCR showing the expression of 1–3, 1–2, and 3 variants from the experiment shown in (**E**). L34 mRNA levels were used for normalization. Bars represent means ± SEM of 4 experiments.

We then analyzed the functional effects of H19 on primary MC epithelial/mesenchymal phenotype. To provide a broader observation, the effect of H19 on the expression of mesenchymal markers was analyzed by both genetic silencing and ectopic expression as a mirror experiment. H19 silencing (all variants) downregulated the expression of mesenchymal markers TGFβRI, SNAIL, PAI-1, and SMAD3, with the exception of SMAD2, which was found increased. On the other hand, the epithelial marker E-Cadherin and the mesothelial differentiation marker WT1 were clearly upregulated (Fig. 2A, B). In line with these results, H19 ectopic expression up-regulated TGFβRI, SNAIL, PAI-1, SMAD2, and SMAD3, while E-Cadherin expression was downregulated at RNA and protein level (Fig. 2C, D). Selective genetic silencing of H19 isoforms revealed that they act synergistically, favoring a mesenchymal phenotype, as revealed by SNAIL, TGFβRI, and E-Cadherin expression (Fig. 2E, F).

**Fig. 3:**
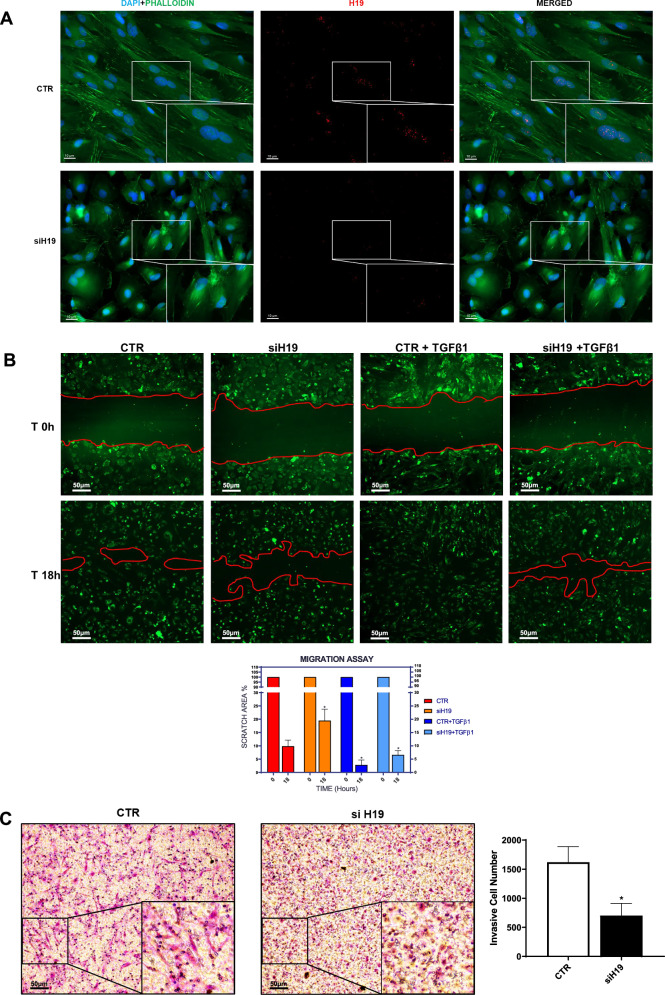
H19 favours MC directed cell migration and invasion through type-1 collagen matrices. **A** Representative image of H19 expression (red), phalloidin (green) in primary MCs by FISH upon H19 silencing. Nuclei (blue) are stained with DAPI. Representative images are shown from one experiment of 3 performed. Scale bar: 10 μm. **B** Effect of H19 genetic silencing on directed cell migration (scratch assay). MCs from patients undergoing PD were allowed to reach 100% confluency in a 12 multiwell plate. MCs were genetically silenced for H19 in the presence of TGFβ1 for 48 h in culture medium supplemented with 10% FCS. Therefore, a scratch was performed with a 10 μl tip and after 18 h cells were fixed and stained with wheat germ agglutinin (green) to stain plasma membrane or Hoechst33342 (blue) to stain nuclei. Representative experiment is shown of three performed. A quantification of the migration assay is shown below. Bars represent means ± SEM of 3 experiments. Scale bar: 50 μm. **C** Effect of H19 genetic silencing on invasion through type I COLLAGEN matrices. A representative experiment is shown one of four performed experiments. Right, histogram resuming the results of the experiment described. Scale bar: 50 μm.

The role of H19 in sustaining a mesenchymal-like phenotype was confirmed by an analysis of cellular morphology and functional assay. Indeed, H19 genetic silencing in mesenchymal-like primary cells from PD patients promoted a reversal towards an epithelial-like cobblestone phenotype and a reorganization of actin stress fibers (Fig. 3A). Accordingly, MC directed migration and invasion on type-1 collagen-coated membranes were impaired by H19 genetic silencing (Fig. 3B, C).

### H19 is downregulated upon HDAC1 inhibition

**Fig. 4:**
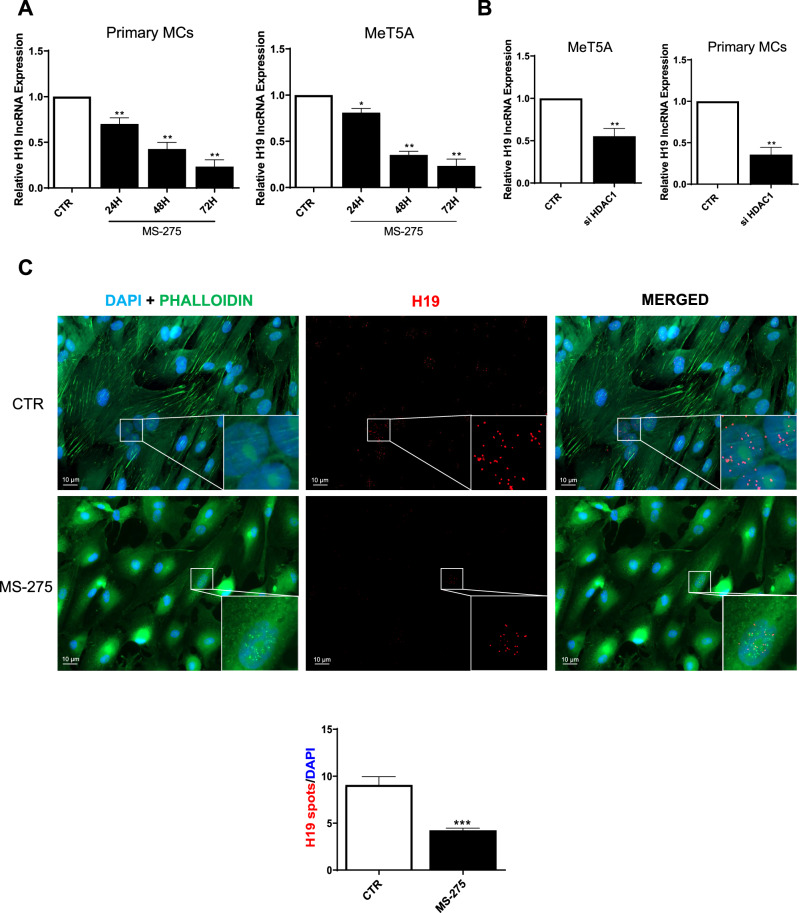
H19 expression is positively controlled by HDAC1. **A** RT-qPCR showing the expression of H19 from MCs from PD patients (left) and MeT5A cells (right) treated with MS-275 (250 nM) for 24, 48, and 72 h. L34 mRNA levels were used for normalization. Bars represent means ± SEM of 4 experiments. **B** RT-qPCR showing the expression of H19, from MCs from PD patients (left) and MeT5A cells (right) genetically silenced for HDAC1. L34 mRNA levels were used for normalization. Bars represent means ± SEM of 4 experiments. **C** Representative image of H19 expression (red), phalloidin (green) in primary MCs by FISH from MCs from PD patients treated with MS-275 (250 nM) for 48 h. A representative experiment is shown one of four performed experiments. Right, histogram resuming the results of the experiment described. Bars represent means ± SEM of 3 experiments. Scale bar: 10 μm.

We then analysed the role of HDAC1 in the modulation of H19 expression. Time course experiments revealed a significant downregulation of H19 in primary MC and MeT5A cells both upon MS-275 treatment and HDAC1 genetic silencing (Figs. 4A, B, S2A). RNA FISH experiments reproduced RT-PCR data (Fig. 4C). These results demonstrate that H19 expression is induced by HDAC1.

### An HDAC1-driven WT1/Sp1 interplay controls H19 expression

**Fig. 5:**
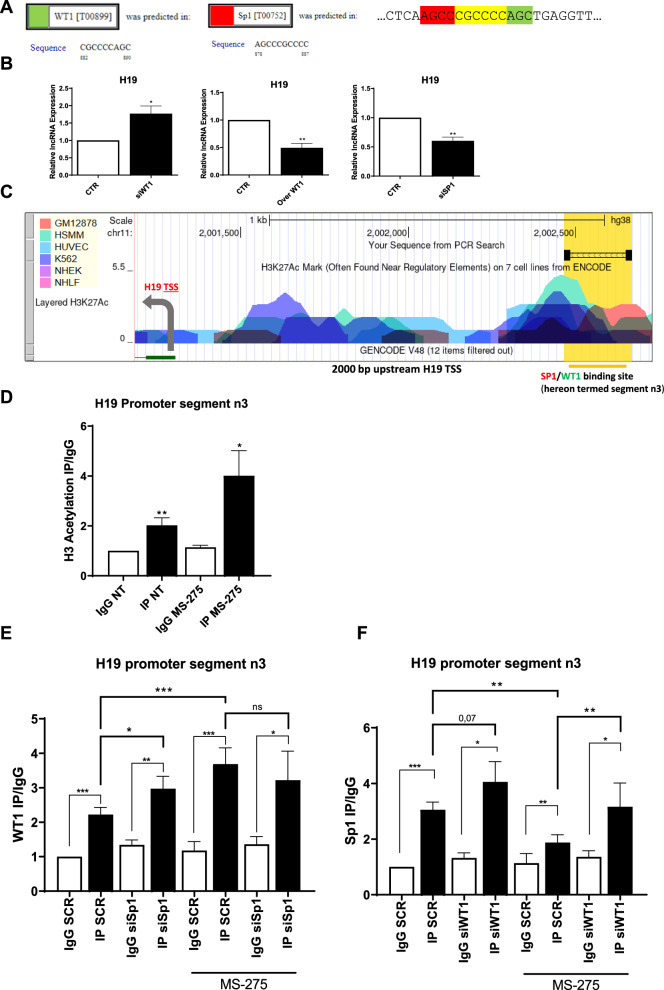
H19 expression is controlled by an HDAC1-mediated WT1/Sp1 switch. **A** Predicted WT1 (left) and Sp1 (middle) binding site on segment n3 on H19 promoter by PROMO. The overlapping sequence is shown in yellow (right). **B** RT-qPCR showing the expression of H19, from MeT5A cells upon WT1 genetic silencing (**B** left) WT1 ectopic expression (**B** middle) and Sp1 genetic silencing (**C**). L34 mRNA levels were used for normalization. Bars represent means ± SEM of 4 experiments. **C** Study of H3K27 acetylation of H19 promoter from ENCODE. WT1/Sp1 binding sites are marked in yellow. **D** Magnetic ChIP experiment showing H3 acetylation on H19 promoter (binding segment n3) using anti-H3Ac IgG and as control, with normal rabbit IgG on chromatin from MeT5A cells treated with DMSO (CTR) or with MS-275. Bars represent means ± SEM of 4 experiment**s**. **E**, **F** ChIP experiment showing WT1 binding (**E**) and Sp1 binding (**F**) on H19 promoter upon treatment of MeT5A cells with MS-275 (250 nM) for 48 h and mutual genetic silencing. Bars represent means ± SEM of 4 experiments.

In order to further shed light on the molecular mechanisms of H19 regulation, a bioinformatic analysis by PROMO of the transcription factors that could bind the H19 promoter was performed, and a binding site for WT1, the master gene of mesothelial differentiation, was found [1, 27] (Fig. 5A left). WT1 expression was previously demonstrated to be induced in a context of HDAC1 inhibition [14]. Interestingly, the WT1 binding sequence on the H19 promoter was partially overlapping the binding site of Sp1, a transcription factor active in the regulation of cellular plasticity previously reported as induced upon exposure to PD fluids in primary MC [28] (Fig. 5A).

The inhibitory role of WT1 on H19 expression was demonstrated by WT1 genetic silencing/ectopic expression. (Figs. 5B, S2B). Conversely, Sp1 genetic silencing demonstrated the role of this transcription factor in favoring H19 expression (Figs. 5C, S2C).

Interestingly, the study of functional elements of the H19 promoter from ENCODE revealed the presence of multiple acetylation peaks overlapping with the predicted H19 binding sites in 6 cell lines analyzed, strongly suggesting a role of histone acetylation/deacetylation in the regulation of H19 expression (Fig. 5D).

As a support for the role of acetylation on this gene regulation, histone 3 (H3) acetylation on WT1/Sp1 binding site was found increased upon treatment of MeT5A cells with MS-275 (Figs. 5E, S3A) in chromatin immunoprecipitation (ChIP) experiments.

The binding of WT1 and Sp1 on the H19 promoter and the impact of HDAC1-mediated histone acetylation were then analyzed. In basal conditions, a specific binding of both WT1 and Sp1 to H19 promoter was demonstrated (Figs. 5F, G, S3B, C). MS-275 treatment favored WT1 while limiting Sp1 binding. The hypothesis of a competition of the two transcription factors on the same binding site was then verified, performing specific genetic silencing.

Indeed, Sp1 genetic silencing significantly increased WT1 binding to H19 promoter in not-treated cells. Interestingly, in MS275-treated conditions, the acetylation is sufficient to cause Sp1 detachment from this region. In a mirror experiment, Sp1 binding was increased upon WT1 genetic silencing both in control and MS275-treated cells suggesting that its recruitment occurs in the absence of WT1 independently of the acetylation status.

**Fig. 6:**
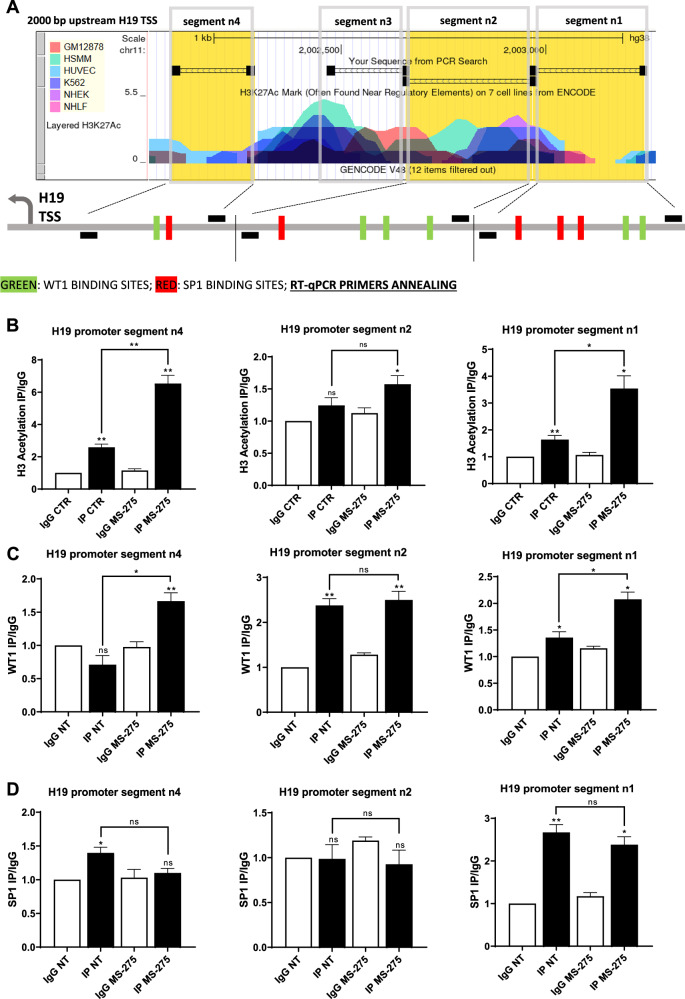
Extension of the analysis of H19 promoter to binding segments n1, n2, and n4. **A** Study of H3K27 acetylation of H19 promoter from ENCODE. The sequence binding segments n4 (left), n2 (middle), and n1 (right) containing predicted WT1 (green) and Sp1 (red) binding sites by PROMO are marked in yellow. **B** Magnetic ChIP experiment showing H3 acetylation on segments n1, n2, and n4 from H19 promoter using anti-H3Ac IgG and normal rabbit IgG as control on chromatin from MeT5A cells treated with DMSO (CTR) or with MS-275. Bars represent means ± SEM of 3 experiment**s**. ChIP experiment showing WT1 (**C**) and Sp1 (**D**) binding on segments n1, n2, and n4 from H19 promoter upon treatment of MeT5A cells with MS-275 (250 nM) for 48 h. Bars represent means ± SEM of 3 experiments.

We extended our analysis to other DNA sequences where putative WT1 and Sp1 promoter binding sites were found by PROMO. Three more segments were analysed (n1, n2, and n4) covering the whole promoter region where putative binding sites for both WT1 and Sp1 were present (Figs. 6A, S4). On each segment, the acetylation profile and the specific binding of WT1 and Sp1 upon treatment with HDAC inhibitor MS-275 were analyzed. As expected, a general trend of increased acetylation upon treatment with MS-275 in all the segments was found, which was not significant in segment n2 (Fig. 6B). When analysing specific binding of WT1, general trend of increased binding upon treatment with MS-275 was found in all the segments, which was not significant in segment n2. In control condition, a basal binding to n4 segment was not found (Fig. 6C). With respect to Sp1, specific binding in segment n2 was not found; in the other two segments, opposite to WT1, we found a general trend of decreased binding upon treatment with MS-275 (Fig. 6C). In brief, these results suggest that other binding sites, with the exception of segment n2 may cooperate with the already described segment n3 in promoting a WT1/Sp1 switch in promoter occupancy upon treatment with MS-275.

## Discussion

In this study, we describe an HDAC1-WT1/Sp1-H19 axis as a novel regulator of MMT and possibly peritoneal fibrosis in MCs from PD patients. Using both human primary MC and a mesothelial cell line, we provide mechanistic evidence that HDAC1 activity promotes H19 expression by favoring a switch between Sp1 and WT1 occupancy of H19 promoter.

While H19 has been extensively studied in different tumors [19–21, 29, 30], few reports analyzed its role non transformed experimental systems, such as endothelial cells, where H19 acted on endothelial cell plasticity modulating the expression of miRNAs or chromatin remodelers [23, 31]. In endometriosis, estrogen favored H19-mediated invasion of eutopic endometrial stromal cells [32].

H19 expression and function in mesothelium have recently been reported in two studies, where the effect of H19 was related to the activity of two antifibrotic drugs, nintedanib and tamoxifen, respectively [24, 25]. In the first study, loss of H19 further strengthened nintedanib-mediated suppressive effects against the MMT process in a mouse model of PF, and a H19–EZH2–KLF2 regulatory axis was elucidated. In the second study, tamoxifen reduced H19 levels by decreasing the ESR1-mediaed transcription of H19 in an experimental system of high glucose-induced peritoneal fibrosis.

In the present study, we first analyzed the effect of H19 on mesothelial plasticity, particularly MMT induction/reversal in primary MCs from PD patients. By performing both H19 genetic silencing and ectopic expression, we found that H19 favors the expression of several mesenchymal markers and their invasive abilities while inhibiting epithelial/mesothelial markers, e.g., E-cadherin and WT1 in MCs. Analysis of the three main isoforms revealed that they act synergistically to favor a mesenchymal phenotype.

The expression of H19 is tightly regulated by epigenetic mechanisms. H19 is regulated by genomic imprinting: normally, H19 is only expressed by the maternal allele due to high methylation of an imprinting control region (ICR) found between H19 and IGF2 in the paternal allele [33]. However, this regulation may be lost in several cancers, leading to biallelic expression [34].

With the aim of further exploring epigenetic mechanisms controlling H19 expression, we focus on the role of class I HDAC inhibition, and in particular on the role of HDAC1 and of the HDAC1-3 pharmacological inhibitor MS-275. Entinostat (MS-275) is a synthetic benzamide specific for class I HDAC and a selective inhibitor for HDAC1-3 [35, 36]. MS-275 activity has been analyzed, especially in a frame of therapy of leukemias and solid tumors in phase II clinical trials.

Previous evidence from our and other laboratories has robustly demonstrated that HDAC activity is central to the execution of a mesenchymal-like program in MCs exposed to pro-inflammatory and pro-fibrotic stimuli. Indeed, decreased histone 3 acetylation was found in mesenchymal-like MCs compared to epithelial-like MCs [13], whereas increased HDAC1 and HDAC2 expression was found in CAFs of mesothelial origin surrounding ovarian metastases in the peritoneum [4].

We have previously documented that HDAC1 inhibition favors the reversal to an epithelial-like phenotype in primary MC due to an effect on Snail transcriptional activity [13]. Additionally, MS-275 was demonstrated to induce several miRNAs, including miR769-5p, directly targeting TGFB-RI and other mesenchymal genes [14]. Moreover, MS-275 was implicated in blocking MC/Ovarian cancer cell interactions due to an effect on β1 integrin activity and ECM production [5].

Here, we found that also H19 expression is markedly inhibited by treatment with MS-275. The role of HDACs and other epigenetic remodellers on H19 expression in pathological conditions has already been analyzed, especially in cancer experimental models. However, HDAC effects appear contextual and cell/stage-specific. In detail, HDAC2 was demonstrated to inhibit H19 expression in colon carcinoma [29]. Accordingly, givinostat, a pan-HDAC inhibitor, favored H19 expression in colorectal cancer cells [30]. Besides HDACs, GSK-J4, a histone lysine demethylase inhibitor, was demonstrated to interrupt an H19/cell adhesion molecules circuitry relevant to prostate cancer metastasis [37].

When analyzing in further detail the role of HDAC in modulating H19 gene expression, we focused on WT1. Upon HDAC1-3 pharmacological inhibition, WT1 expression was increased, as previously demonstrated [14].

An inhibitory role of WT1 on H19 expression was demonstrated by WT1 genetic silencing/ectopic expression. Moreover, we demonstrated by ChIP assays that WT1 directly binds the H19 promoter. To our knowledge, this inhibitory effect of WT1 on H19 expression has never been described elsewhere. We hypothesized that WT1, whose interaction with the H19 promoter is increased by HDAC1-3 inhibition, may have an inhibitory effect interfering with the binding of other activatory transcription factors. Conversely, Sp1 was demonstrated to favor H19 expression. Sp1 has been described to induce H19 expression, and its putative binding site to the H19 promoter overlaps the WT1 binding site, according to bioinformatic analysis. Notably, ChIP for both WT1 and Sp1 in interfered cells disclosed the mutually exclusive binding of these factors to H19 promoters and its dependence on the chromatin acetylation status.

How may HDACs regulate WT1/Sp1 expression? According to recent literature, HDACs may regulate the competitive binding of SP1 and WT1 through distinct mechanisms. SP1 is a direct target of HDAC-mediated deacetylation. Specifically, HDAC1, HDAC2, HDAC 6 and HDAC10 deacetylate SP1 at lysine 703, a modification that stabilizes its DNA-binding capacity and prevents degradation. This has been demonstrated in non-small cell lung cancer and glioblastoma cells, where HDAC inhibition increases SP1 acetylation, reduces its binding to target promoters, and suppresses downstream gene expression [38, 39]. Interestingly, these results linking histone increased acetylation with reduced binding to target promoter are in line with our experimental evidence. Conversely, WT1 seems not to be significantly regulated by direct acetylation. Instead, HDACs modulate WT1 activity indirectly via chromatin remodeling. HDAC inhibitors such as TSA and SAHA in distinct hematologic cell lines induce hyperacetylation at intronic enhancer elements of the WT1 gene, leading to reduced expression and transcriptional activity of the WT1 protein [40]. Thus, we found a discrepancy between these published results and our experimental evidence, possibly linked to different HDAC inhibitors used and to cell specificity. In summary, we have identified a novel regulatory pathway mediated by the HDAC1-WT1/Sp1-H19 axis that modulates the myofibroblastic phenotype in primary MC from PD patients. The fact that H19 has been found in EVs further increases the potential role of this lncRNA both in cell autonomous and non-autonomous regulation of cellular plasticity in different experimental systems [41]. Growing capacity to exploit RNA-based approaches shall benefit from basic science reports exploring the ability of specific ncRNAs to modulate cellular plasticity in order to counteract peritoneal fibrosis.

## Supplementary information

Supplementary figure legends

Figure S1

Supplementary Figure 2

Supplementary Figure 3

Supplementary Figure 4

## Data Availability

All datasets generated and analysed during this study are included in this published article and its Supplementary Information files. Additional data are available from the corresponding author on reasonable request.
