# Supplementary material for: Long noncoding RNA H19 promotes the acquisition of a mesenchymal-like invasive phenotype in mesothelial primary cells through an HDAC1-mediated WT1/Sp1 switch
Source: Cell Death Dis. 2025 Aug 31;16(1):663. doi: 10.1038/s41419-025-07956-8 (PMC12398590; doi:10.1038/s41419-025-07956-8)
Supplement: Supplementary file 1 — Supplementary figure legends [file 41419_2025_7956_MOESM1_ESM.docx]

**Supplementary Figure legends**

**Supplementary Figure 1.** Raw data of WB shown in this study.

**Supplementary Figure 2.** RT-qPCR showing the expression of HDAC1, WT1 and Sp1 in MeT5A cells from the experiments shown in Fig. 4B, 5A and 5B, respectively. L34 mRNA levels were used for normalization. Bars represent means±SEM of 4 experiments

**Supplementary Figure 3.** Negative amplification ChIP control of Human RPL30 promoter upon MS-275 and genetic silencing of WT1 and SP1 from the experiment shown in Fig. E-G.

**Supplementary Figure 4.** Top, study of predicted WT1 (green) and Sp1 (red) binding sites on H19 promoter by PROMO. TATA box is marked in purple. Bottom, qPCR primers used to amplify segments 1, 2, 3 and 4 are shown.
