# Supplementary material for: Long noncoding RNA H19 promotes the acquisition of a mesenchymal-like invasive phenotype in mesothelial primary cells through an HDAC1-mediated WT1/Sp1 switch
Source: Cell Death Dis. 2025 Aug 31;16(1):663. doi: 10.1038/s41419-025-07956-8 (PMC12398590; doi:10.1038/s41419-025-07956-8)
Supplement: Supplementary file 2 — Figure S1 [file 41419_2025_7956_MOESM2_ESM.pptx]

## Slide 1
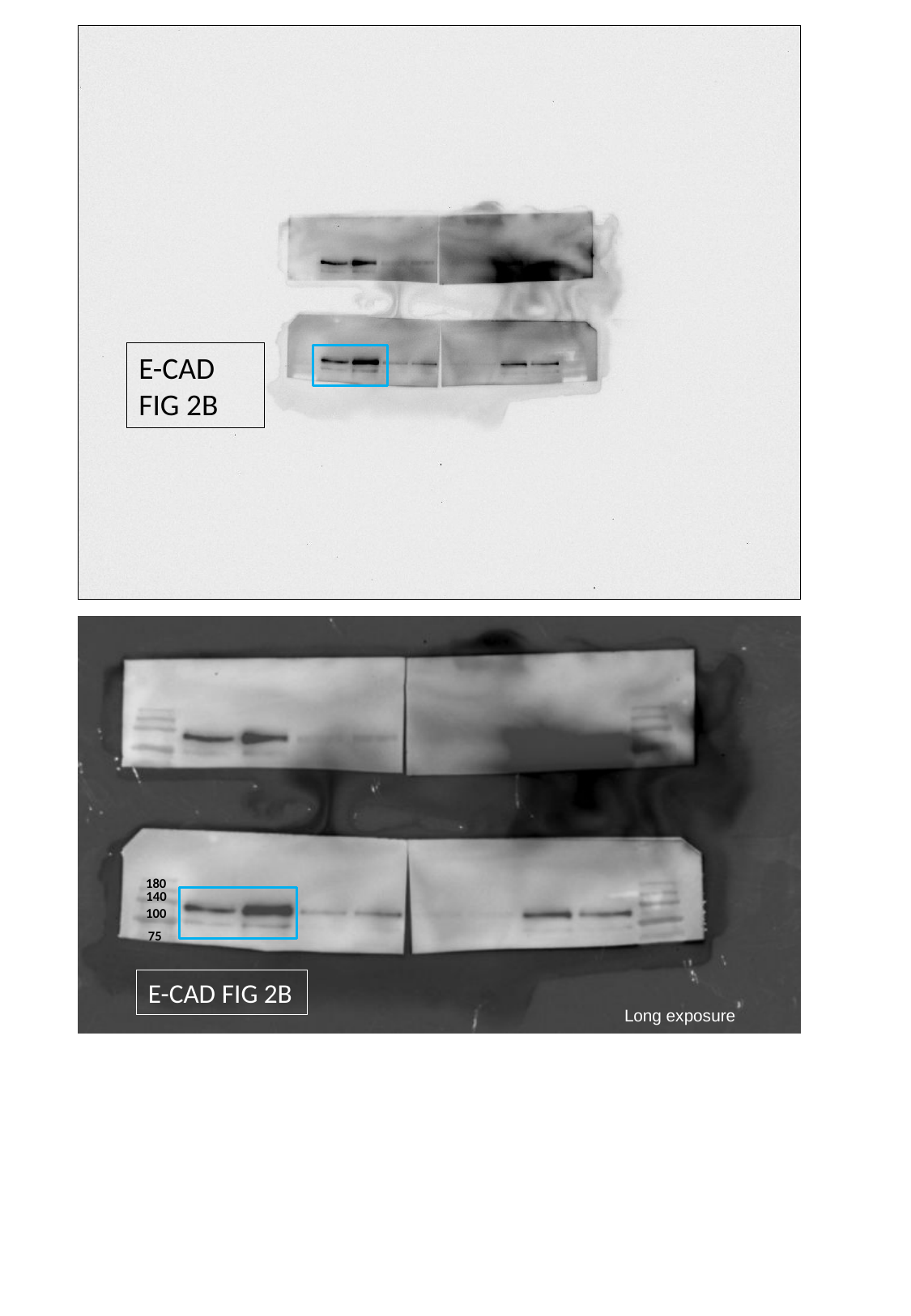

E-CAD
FIG 2B
180
140
100
75
E-CAD FIG 2B
Long exposure

## Slide 2
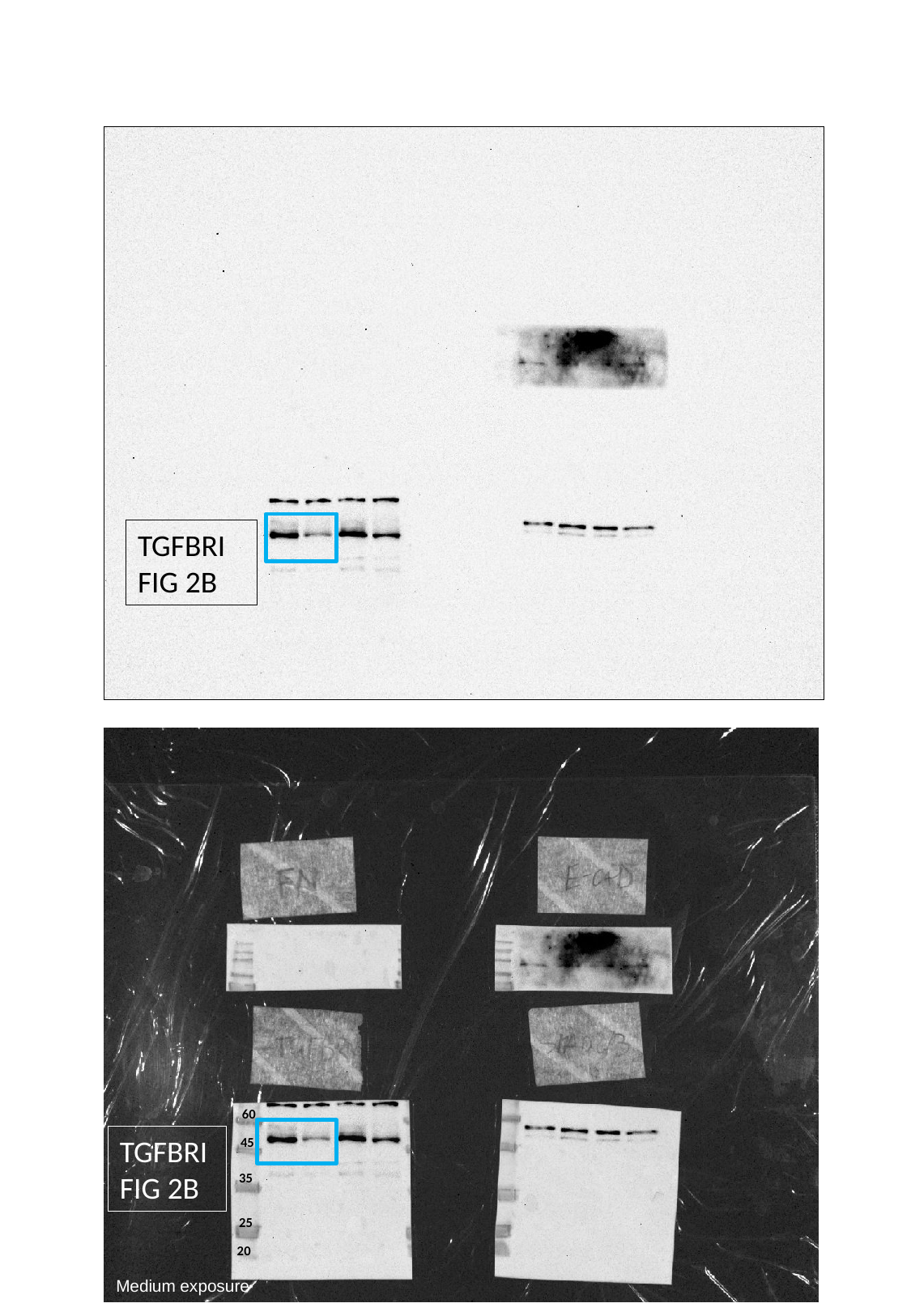

TGFBRI FIG 2B
60
TGFBRI FIG 2B
45
35
25
20
Medium exposure

## Slide 3
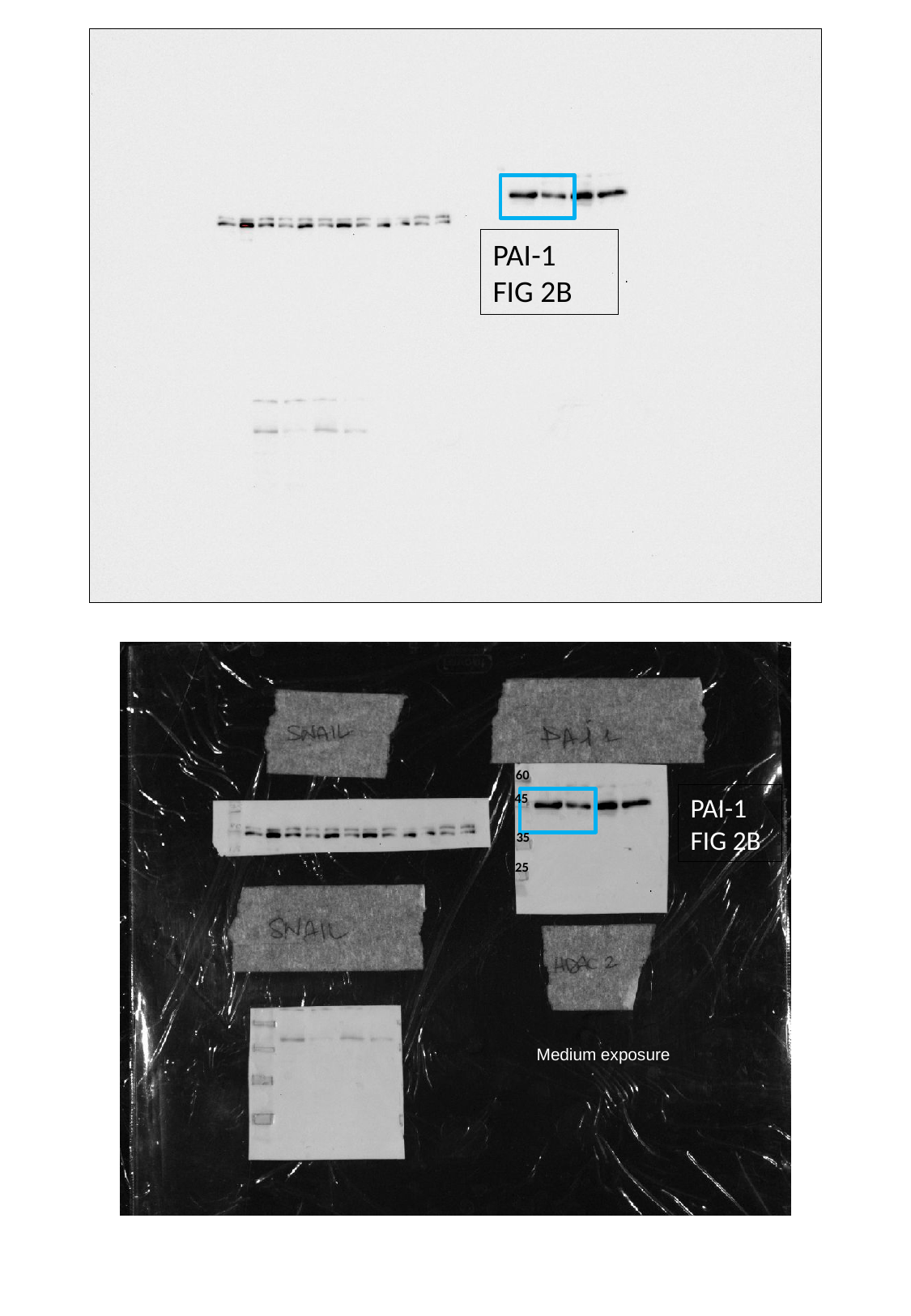

PAI-1
FIG 2B
60
45
PAI-1
FIG 2B
35
25
Medium exposure

## Slide 4
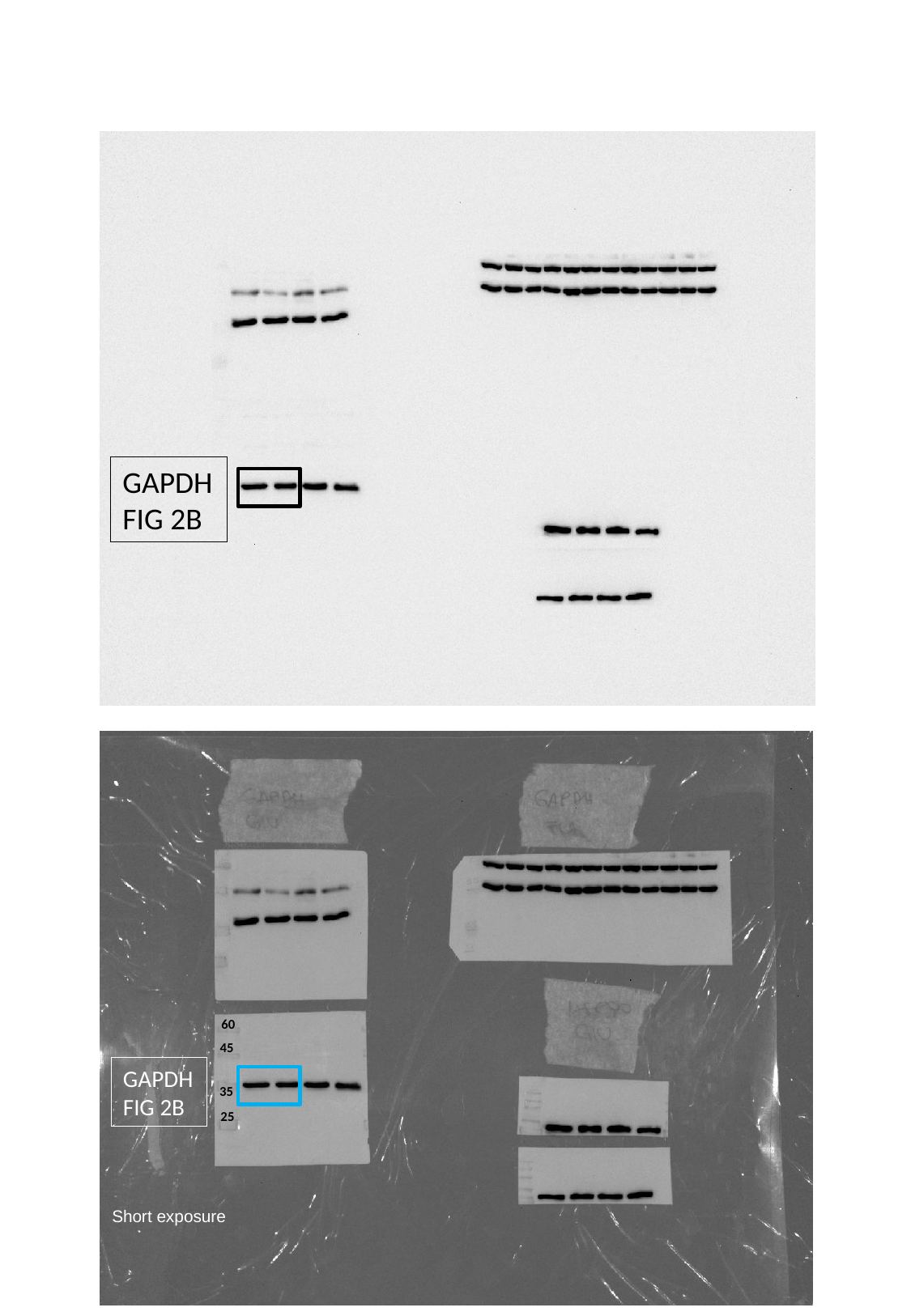

GAPDH
FIG 2B
60
45
GAPDH
FIG 2B
35
25
Short exposure

## Slide 5
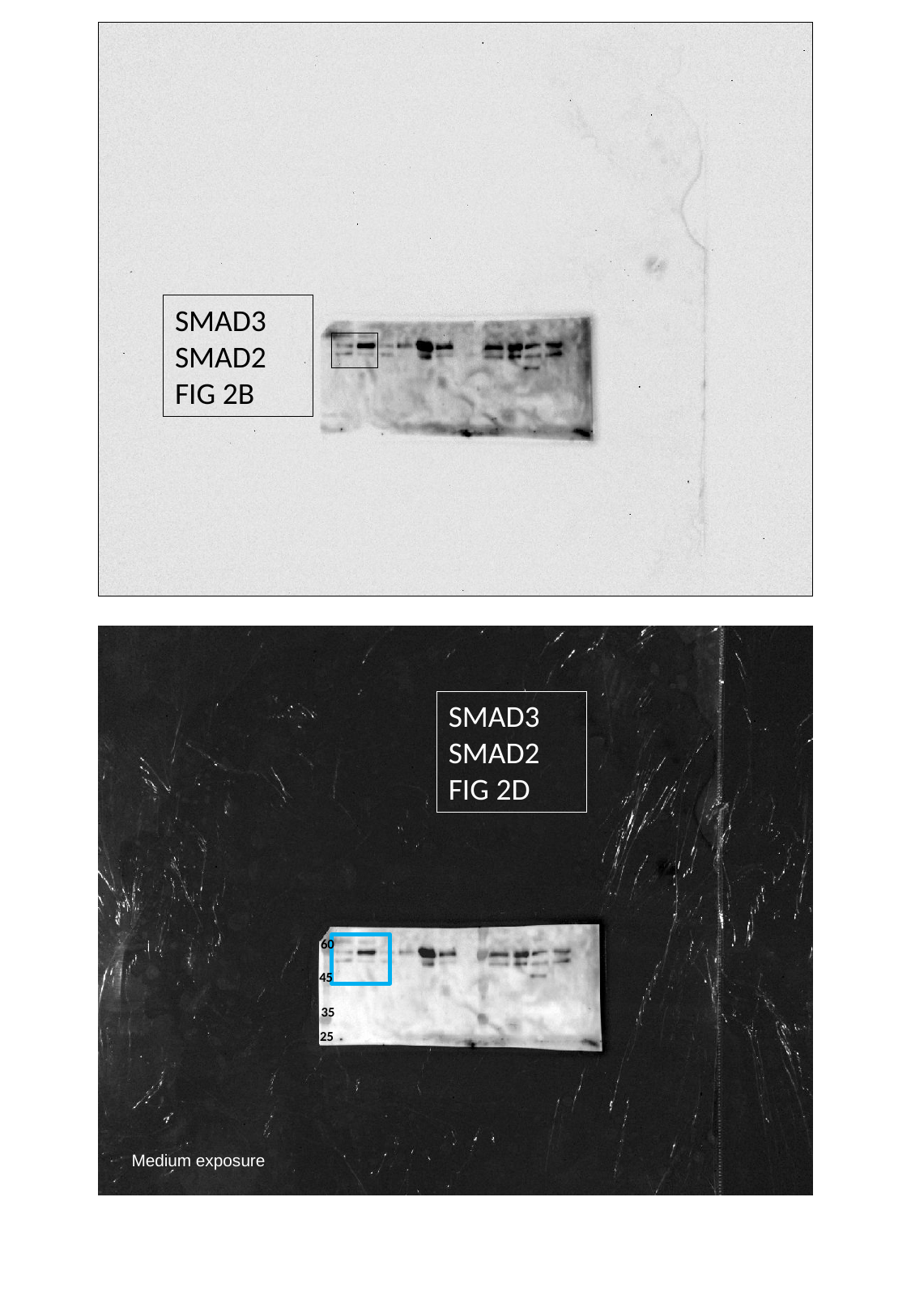

#
SMAD3
SMAD2
FIG 2B
SMAD3
SMAD2
FIG 2D
60
45
35
25
Medium exposure

## Slide 6
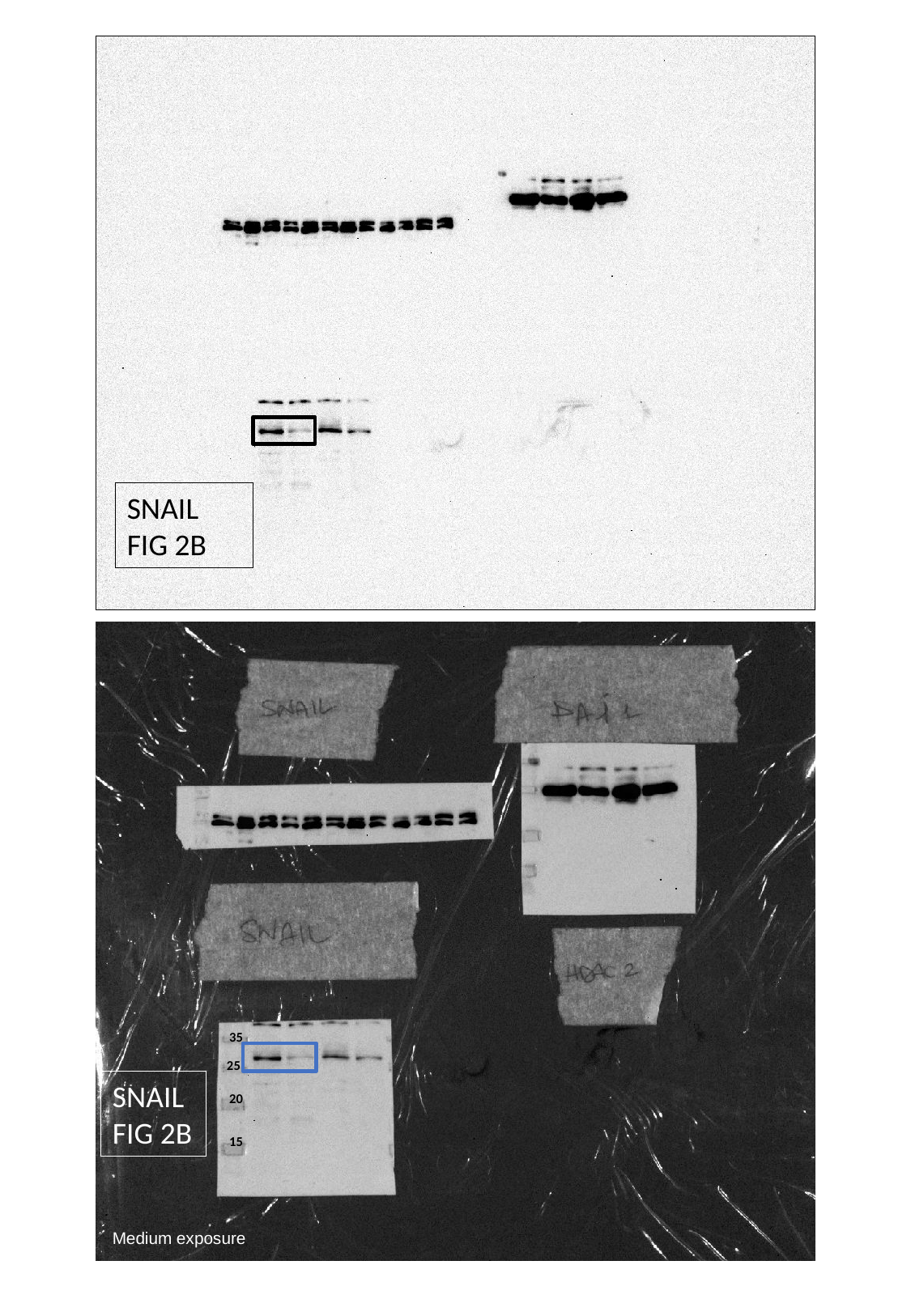

SNAIL
FIG 2B
35
25
SNAIL
FIG 2B
20
15
Medium exposure

## Slide 7
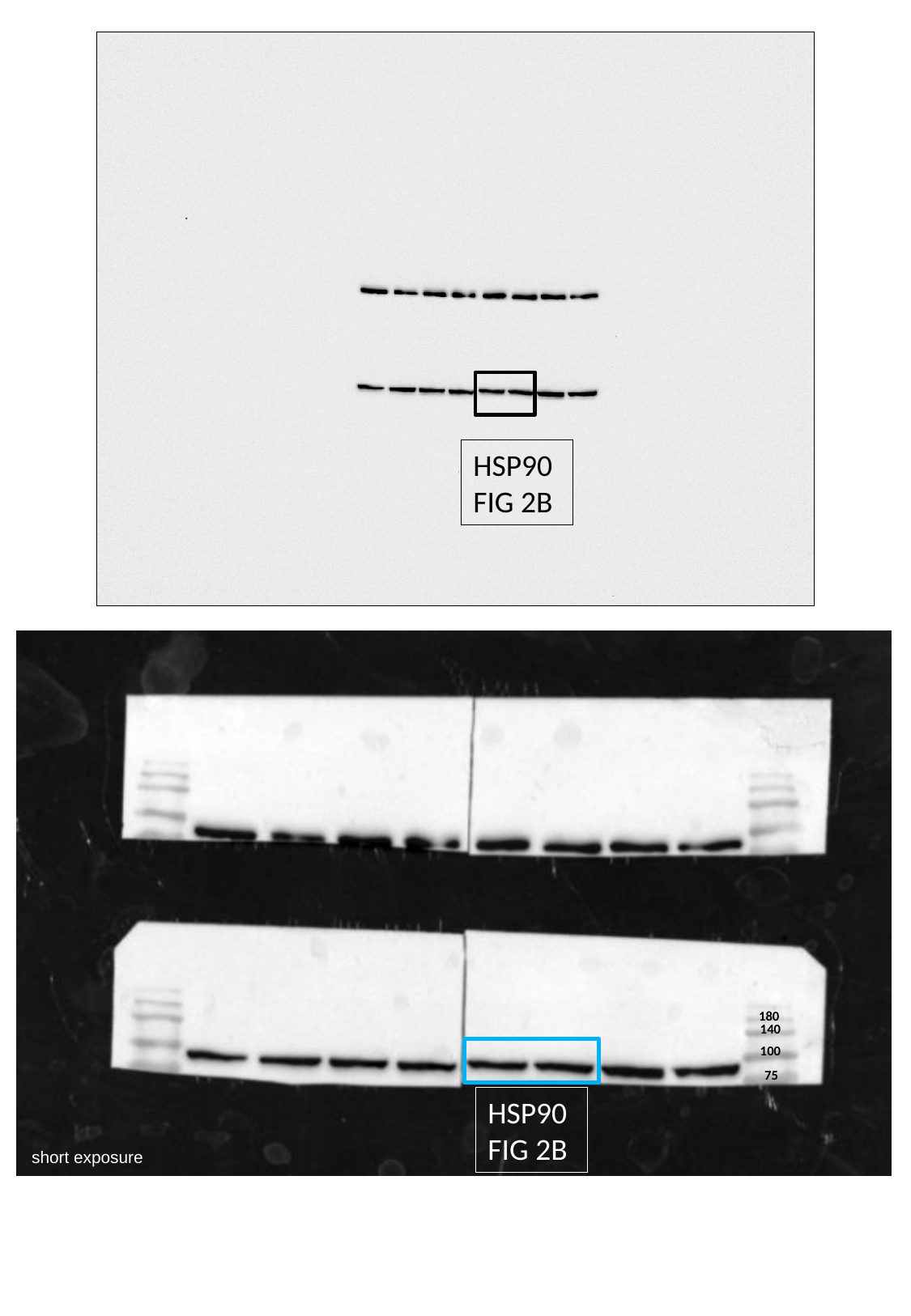

HSP90
FIG 2B
180
140
100
75
HSP90
FIG 2B
short exposure

## Slide 8
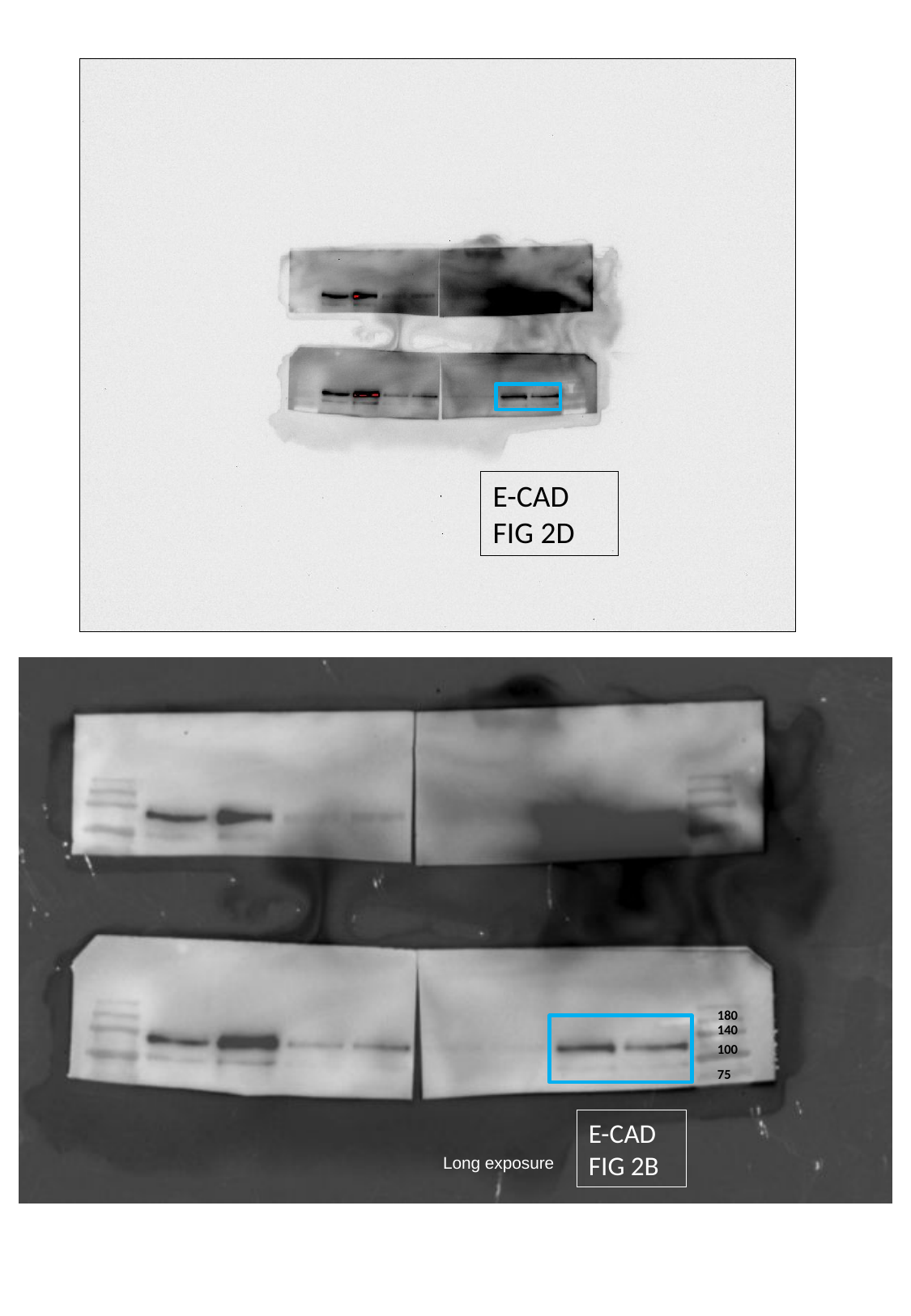

E-CAD
FIG 2D
180
140
100
75
E-CAD
FIG 2B
Long exposure

## Slide 9
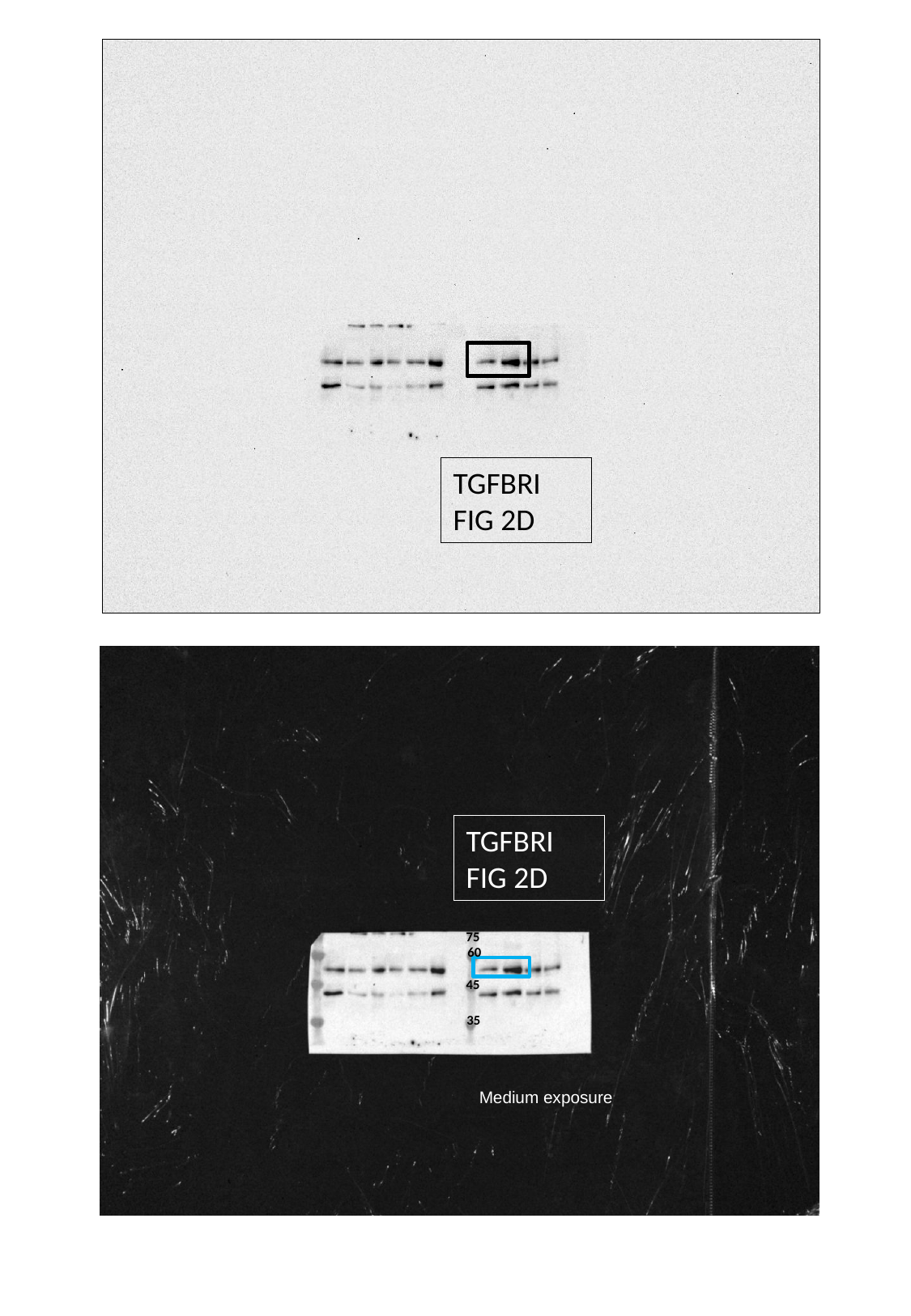

TGFBRI
FIG 2D
TGFBRI
FIG 2D
75
60
45
35
Medium exposure

## Slide 10
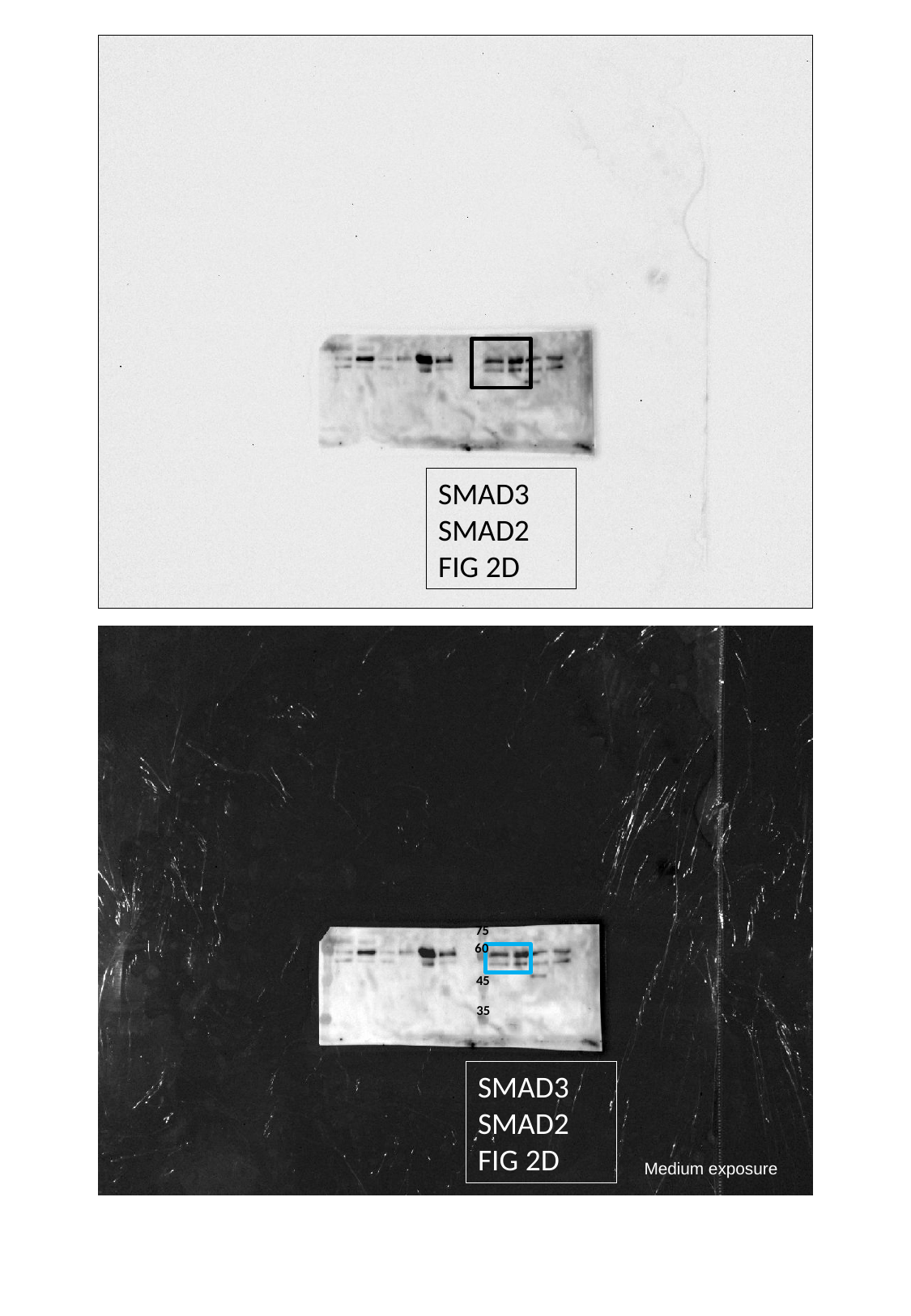

SMAD3
SMAD2
FIG 2D
75
60
45
35
SMAD3
SMAD2
FIG 2D
Medium exposure

## Slide 11
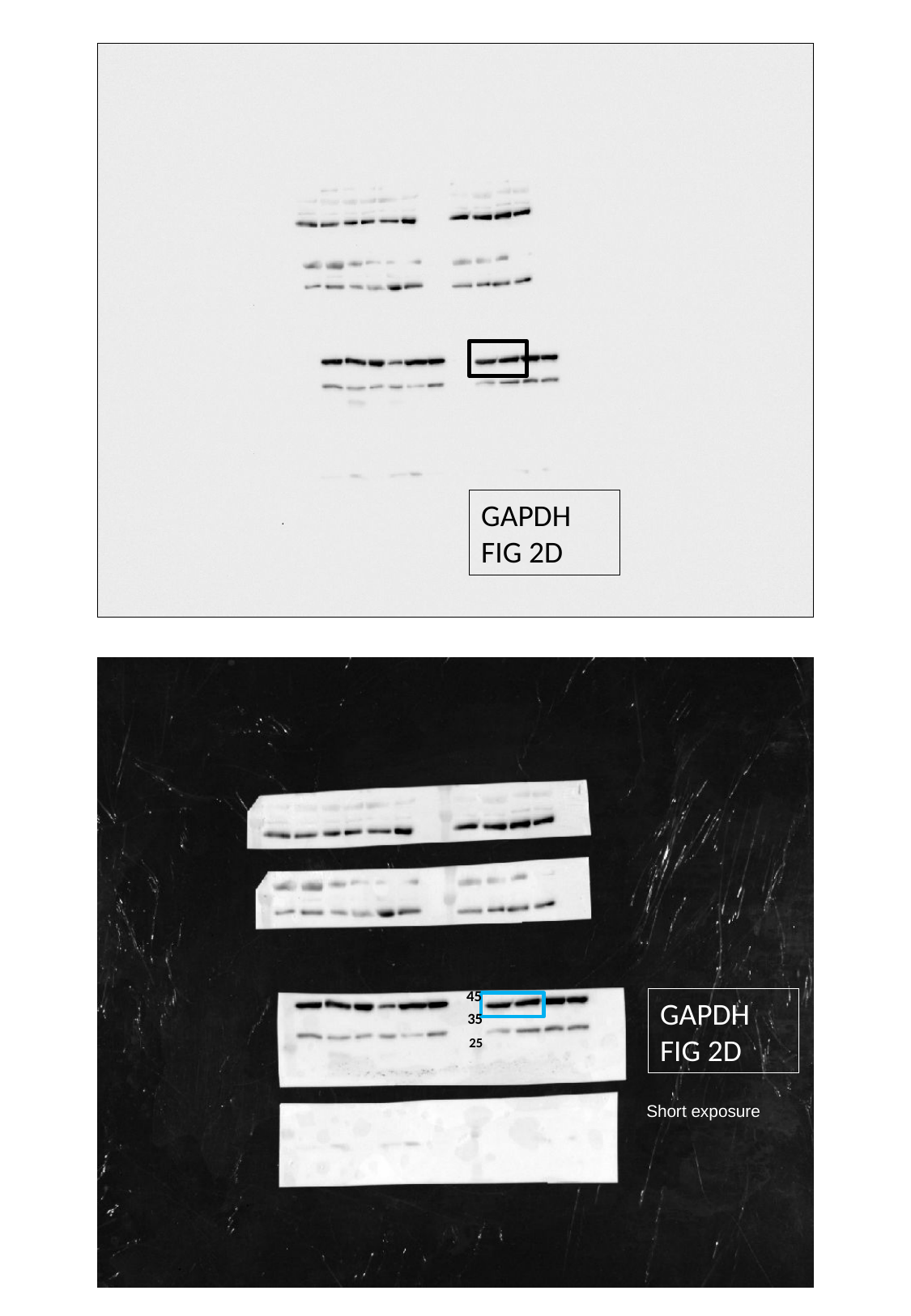

GAPDH
FIG 2D
45
GAPDH
FIG 2D
35
25
Short exposure

## Slide 12
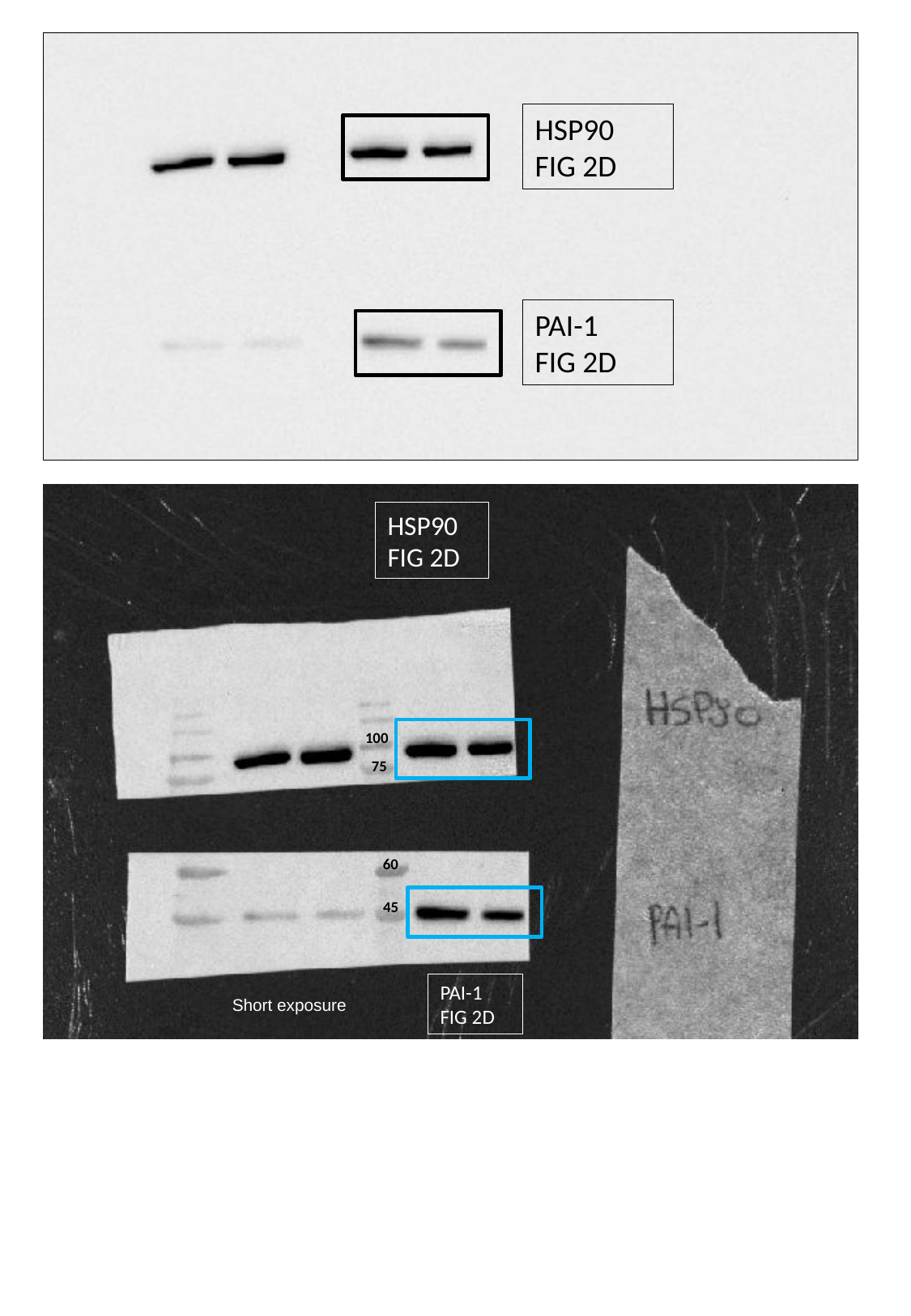

HSP90
FIG 2D
PAI-1
FIG 2D
HSP90
FIG 2D
100
75
60
45
PAI-1
FIG 2D
Short exposure

## Slide 13
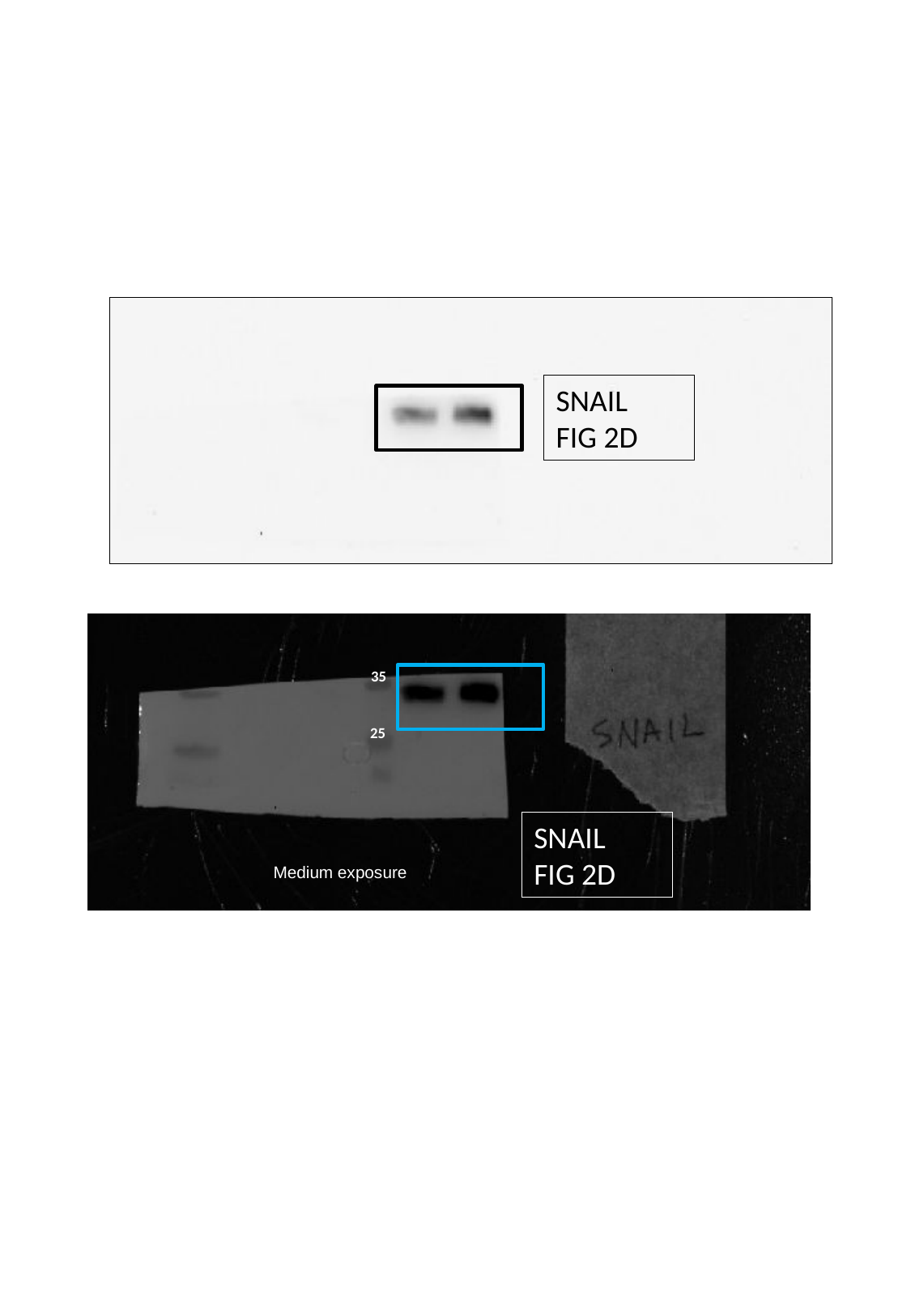

SNAIL
FIG 2D
35
25
SNAIL
FIG 2D
Medium exposure
