# Supplementary figures and images for: Long noncoding RNA H19 promotes the acquisition of a mesenchymal-like invasive phenotype in mesothelial primary cells through an HDAC1-mediated WT1/Sp1 switch
Source: Cell Death Dis. 2025 Aug 31;16(1):663. doi: 10.1038/s41419-025-07956-8 (PMC12398590; doi:10.1038/s41419-025-07956-8)

## Slide 1
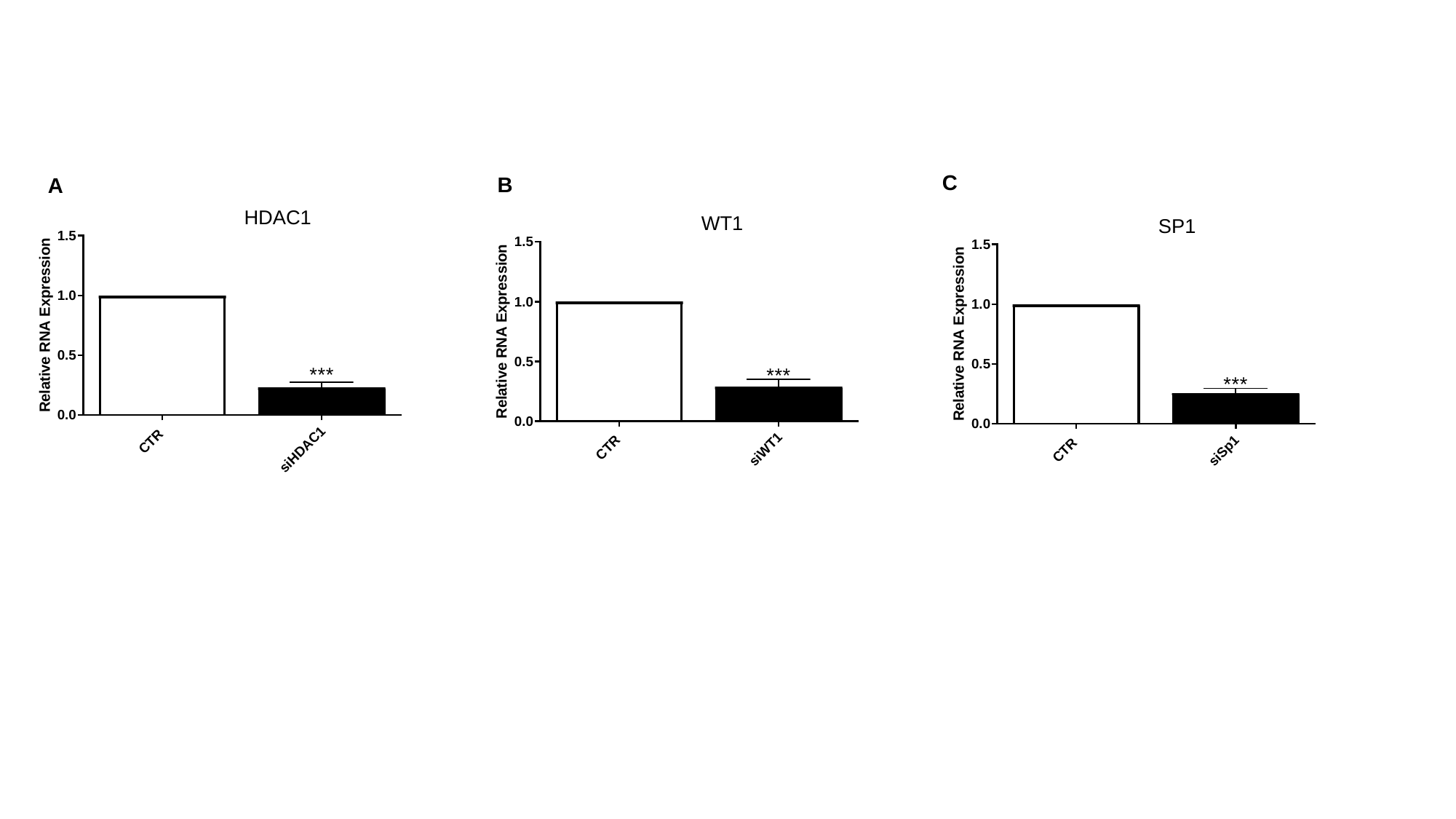

C
B
A

Supplement: Supplementary file 3 — Supplementary Figure 2 [file 41419_2025_7956_MOESM3_ESM.pptx]

## Slide 1
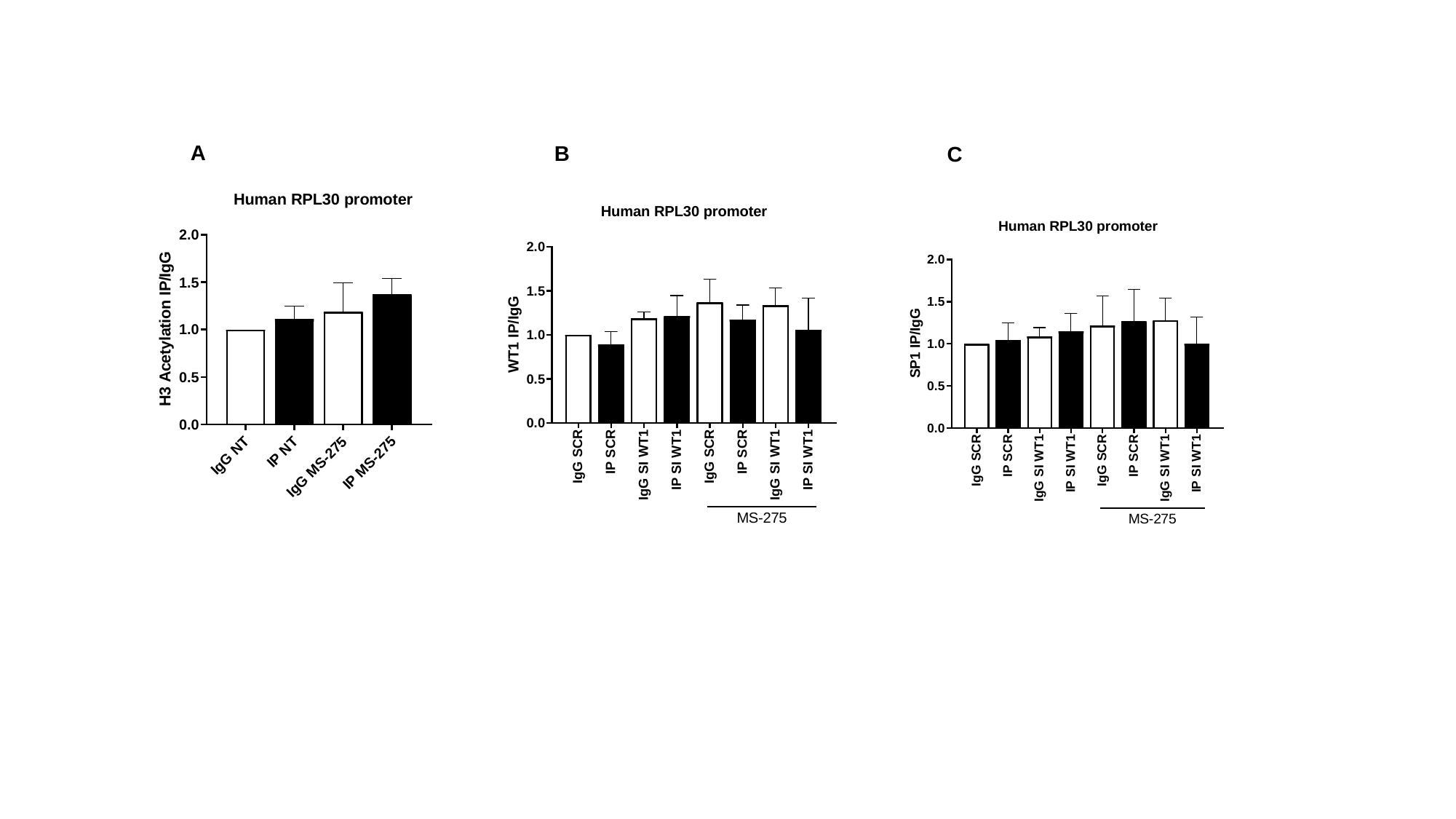

A
B
C

Supplement: Supplementary file 4 — Supplementary Figure 3 [file 41419_2025_7956_MOESM4_ESM.pptx]

## Slide 1
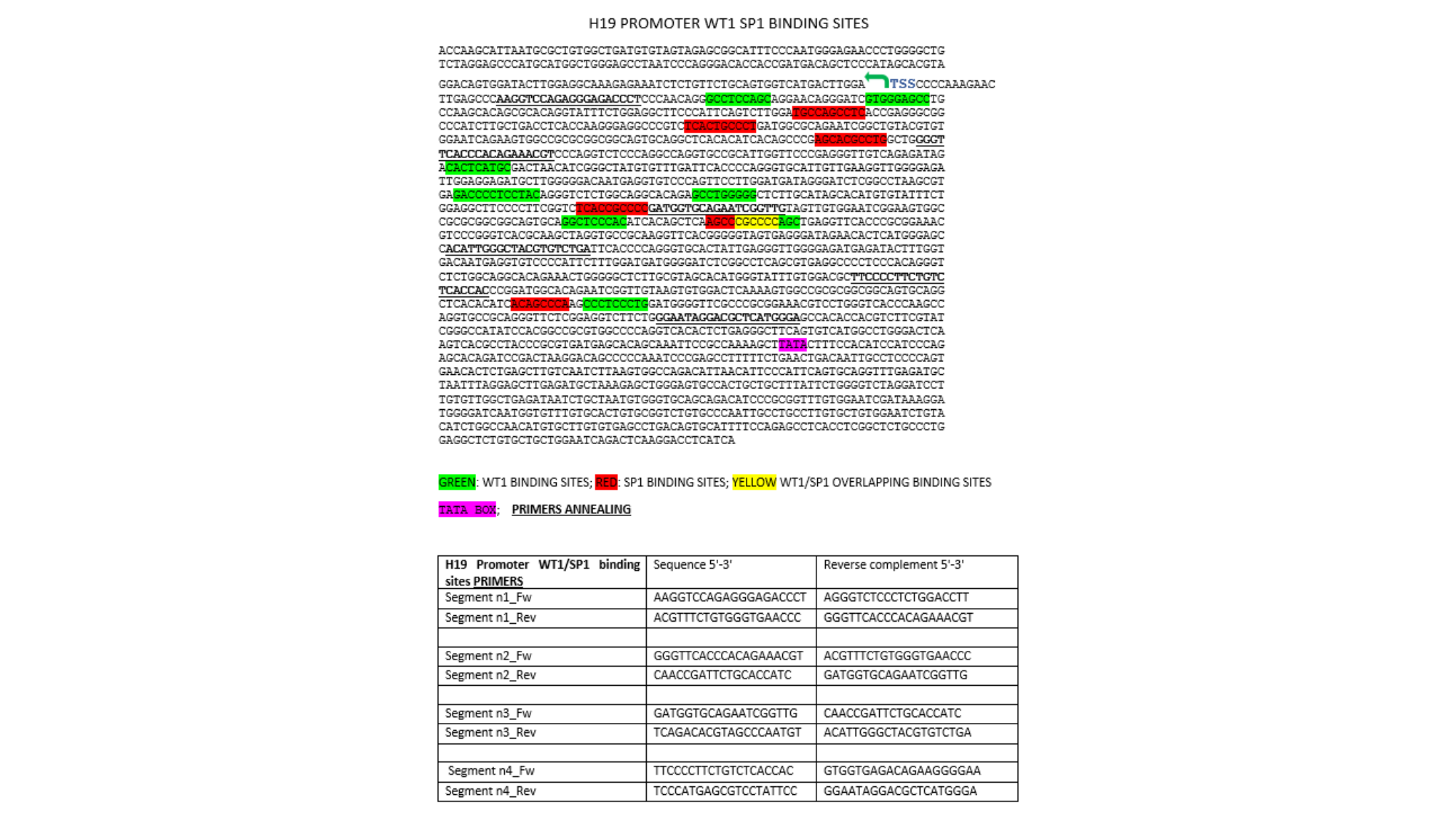

Supplement: Supplementary file 5 — Supplementary Figure 4 [file 41419_2025_7956_MOESM5_ESM.pptx]
